# Gamma oscillations in somatosensory cortex recruit prefrontal and descending serotonergic pathways in aversion and nociception

**DOI:** 10.1038/s41467-019-08873-z

**Published:** 2019-02-28

**Authors:** Linette Liqi Tan, Manfred Josef Oswald, Céline Heinl, Oscar Andrés Retana Romero, Sanjeev Kumar Kaushalya, Hannah Monyer, Rohini Kuner

**Affiliations:** 1 Pharmacology Institute, Medical Faculty Heidelberg, Im Neuenheimer Feld 366, 69120 Heidelberg, Germany; 2 Department of Clinical Neurobiology, Medical Faculty Heidelberg and German Cancer Research Center, Im Neuenheimer Feld 280, 69120 Heidelberg, Germany

## Abstract

In humans, gamma-band oscillations in the primary somatosensory cortex (S1) correlate with subjective pain perception. However, functional contributions to pain and the nature of underlying circuits are unclear. Here we report that gamma oscillations, but not other rhythms, are specifically strengthened independently of any motor component in the S1 cortex of mice during nociception. Moreover, mice with inflammatory pain show elevated resting gamma and alpha activity and increased gamma power in response to sub-threshold stimuli, in association with behavioral nociceptive hypersensitivity. Inducing gamma oscillations via optogenetic activation of parvalbumin-expressing inhibitory interneurons in the S1 cortex enhances nociceptive sensitivity and induces aversive avoidance behavior. Activity mapping identified a network of prefrontal cortical and subcortical centers whilst morphological tracing and pharmacological studies demonstrate the requirement of descending serotonergic facilitatory pathways in these pain-related behaviors. This study thus describes a mechanistic framework for modulation of pain by specific activity patterns in the S1 cortex.

## Abstract (web summary)

Gamma oscillations in somatosensory areas in humans correlate with pain perception and pain stimulus intensity, but could also reflect cognitive processes such as attention. Here the authors provide evidence in mice that these oscillations causally contribute to pain perception.

## Introduction

The nature of circuits and activity patterns underlying the perception of pain is still unknown, and understanding how these change over the course of pain chronicity remains a challenge^1–3^. Rhythmic oscillatory activity in cortical circuits is the cornerstone of cortical function and there has been an increasing interest in understanding cortical activity rhythms in pain^4–7^. In landmark studies on human subjects, pain-related oscillatory activity at higher gamma frequencies (>40 Hz) in the somatosensory S1 cortex was reported to match in amplitude to the objective stimulus intensity as well as the subjective pain intensity^5,8–10^. However, several critical functional and mechanistic aspects remain to be resolved^6^. Importantly, owing to the limited ability for interventional manipulations in humans, it remains unclear whether neuronal synchronization in the gamma range functionally directly impacts on nociception and pain or whether it is only indirectly involved, or even just constitutes an epiphenomenon. Gamma oscillations can occur within the cerebral cortex during many cognitive processes such as attention, learning, diverse types of memory etc.^4^, thereby raising the question whether they are causally linked to pain perception or only unspecifically so, for example, via the modulation of attention^6^. Notably, very little is known so far about the nature of circuits modulated by cortical gamma activity, and their functional contributions towards pain. Oscillatory activity in other frequency bands, such as theta, has also been linked to pain states in human subjects^6^. Building upon previous research^11–13^, we therefore reasoned that an unbiased analyses of activity across frequency ranges in acute nociception and persistent pain states in mouse models would enable testing functional significance of diverse oscillatory rhythms.

GABAergic interneurons, particularly of the fast-spiking parvalbumin type (PV), are important determinants of the integrity of synchronous activity patterns in the brain^14–18^. Consequently, optogenetically-induced rhythmic firing of PV neurons can entrain a gamma rhythm by synchronizing the firing of excitatory (pyramidal) neurons in the S1 barrel cortex^14,19^. Interestingly, PV neurons have been also linked to the generation of theta rhythms in the hippocampus^20^ and neocortex^21^.

Here, we recorded and manipulated diverse activity rhythms in the S1 cortex of awake, behaving mice and report direct functional links to pain-associated behaviors, thus establishing their validity for testing these key questions. We report that among diverse oscillatory rhythms, only gamma range activity was significantly enhanced specifically upon noxious stimulation. Inflamed mice demonstrated hypersensitivity to normally innocuous stimuli, which elicited enhanced gamma power only in inflamed mice. By using optogenetic activation of PV neurons to induce frequency-specific oscillations selectively in the mouse hindlimb S1 cortex, we demonstrate that increased gamma power, but not activity over other frequency bands, potentiates behavioral sensitivity to nociceptive stimuli and induces aversion independently of involvement or modulation of motor activity or attention. Using activity mapping, tracing and pharmacological manipulations in behaving mice, we report the nature of cortical and subcortical centers involved and demonstrate that gamma activity in the S1 recruits descending serotonergic pathways originating in the raphe magnus nucleus to facilitate nociceptive sensitivity.

## Results

### Increased gamma power in the S1 cortex during nocifensive behavior

**Fig. 1:**
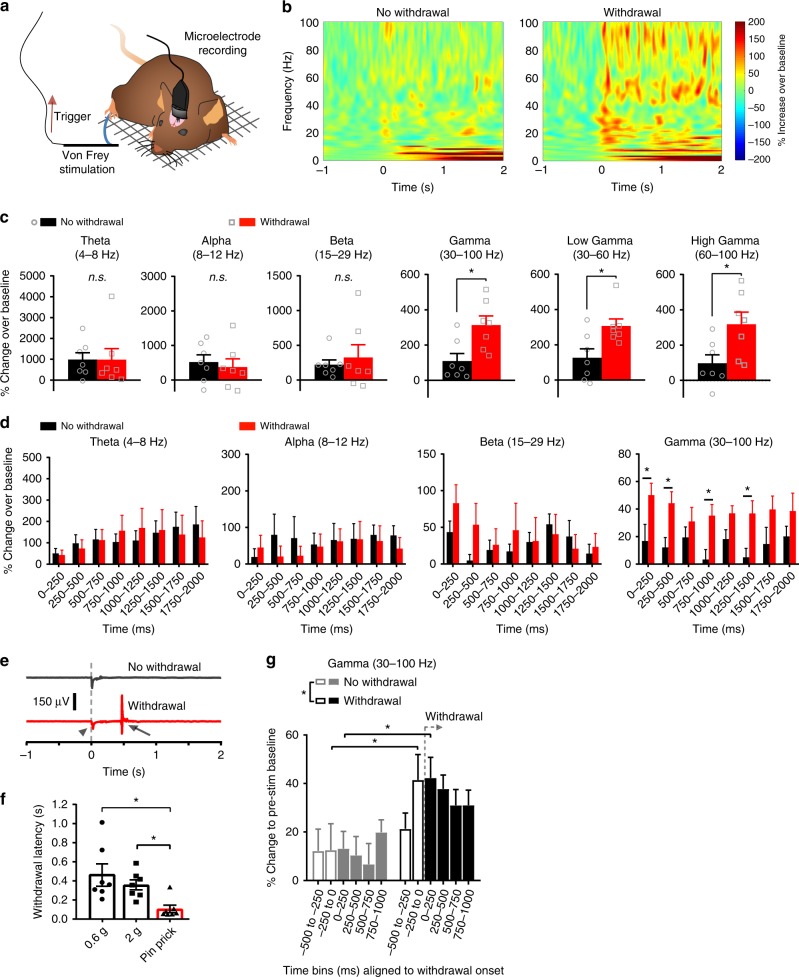
Increased gamma-band oscillatory power in the S1 hindlimb cortex (S1HL) of mice in conjunction with nocifensive behavior. **a** Scheme of the experimental procedure. Animals were sitting on a grid while receiving triggered von Frey stimulation on the hindpaw contralateral to implantation. **b** Left: Time-frequency representation of spectral modulation in S1HL for all trials with no paw withdrawal in response to 2 g von Frey stimulation of the contralateral hindpaw. Right: Time-frequency representation of the same animals (*n* = 7) in withdrawal trials in response to 2 g von Frey filaments (grand mean, 5–7 applications per filament and animal). Power is coded as event-related perturbation (ERP) representing the deviation from the mean over a 1000 ms baseline period immediately preceding stimulus onset. **c**, **d** Quantification of the time-frequency representations shown in **b** in the absence and presence of paw withdrawals to applications of 2 g filaments for different frequency bands over the 2 s post-application period. Data was averaged first over all withdrawal and no-withdrawal trials over the specified frequency ranges in 250 ms time bins for each animal and are plotted as **c** the cumulative ERP (*n* = 7; *p < 0.05, Student’s paired *t*-test) or **d** the binned and time-resolved ERP (*n* = 7; *p < 0.05, two-way repeated measures ANOVA with Bonferroni multiple comparison test). **e** Representative piezo transducer signal episodes during the application of a 2 g von Frey filament without (top) and with (bottom, red) a paw withdrawal. Stimulus onset (arrowhead) and offset (arrow) at the start of the negative and positive deflections, respectively, are marked. **f** Median paw withdrawal latencies in response to 0.6 g, 2 g von Frey filaments or a pin-prick (*n* = 7; *p < 0.05, one-way repeated measures ANOVA with Tukey’s multiple comparisons test). **g** Comparison of gamma-band ERP in % power aligned to the withdrawal onset and averaged over 250 ms time bins for trials without (gray) and with (black) paw withdrawals to applications of 2 g von Frey filaments (*n* = 7; *p < 0.05, two-way repeated measures ANOVA with Bonferroni multiple comparison test). Data are represented as mean ± S.E.M.

We recorded field potentials and network oscillatory activity in freely moving mice via microelectrodes that were chronically implanted in the hindlimb representation region of the mouse S1 (S1HL, Fig. 1a). Using von Frey filaments, we applied 2 g punctate mechanical force to the plantar hindpaw contralateral to the S1HL, in which activity recordings were simultaneously performed. A 2 g stimulus is considered noxious in mice, based upon previous behavioral studies and is sufficient to activate a majority of C- and A-fiber mechano-nociceptors in electrophysiological studies^22–26^. Here, mice chronically implanted with cortical microelectrodes demonstrated somewhat higher thresholds than the typical values of 0.6–1 g that have been reported as the 50% noxious threshold in C57Bl6 mice^23,27^. Therefore, although mice typically demonstrated withdrawal behavior, some trials of 2 g applications also resulted in lack of withdrawal. As compared to pre-application baseline, noxious mechanical stimulation resulted in increased activity across diverse frequency ranges (Fig. 1b). However, unlike activity in the theta (4–8 Hz), alpha (8–12 Hz), beta (15–29 Hz) ranges, activity in the gamma frequency range (i.e., 30–100 Hz) in the S1HL was increased to a significantly higher extent in those trials for which mice demonstrated a withdrawal to von Frey stimulation as compared to trials for which mice did not withdraw their paw away from the same mechanical stimulus (Fig. 1c). This included both low range gamma (30–60 Hz) as well as high range gamma (60–100 Hz) (Fig. 1c and Supplementary Fig. 1). To facilitate identification of time-frequency locations at which significant differences between the two groups become apparent, we broke down the data on diverse frequency bands into 250 ms time bins and observed that unlike theta, beta and alpha activity, increased gamma power was evident immediately following stimulus onset, with significant increase within the first 250 ms following stimulus onset (Fig. 1d). This signal in the S1 temporally preceded withdrawal, since the mean withdrawal latency for the 2 g filament was measured to be 358 ± 52 ms in the same mice (Fig. 1e, f). When we re-analyzed the same data upon setting the onset of the withdrawal behavior as time zero, it was evident that enhancement of gamma activity in the S1 cortex preceded the behavioral nocifensive reactions to the noxious stimulus (Fig. 1g).

### Inflamed mice have increased gamma oscillatory power in the S1 cortex

**Fig. 2:**
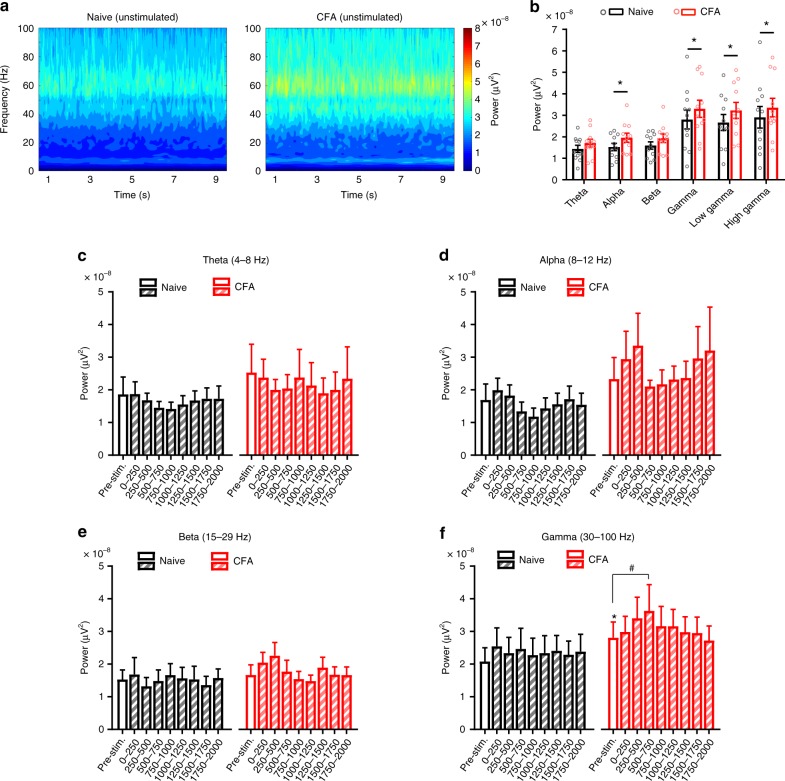
Mice with ongoing paw inflammation have increased gamma oscillatory power in the S1 hindlimb cortex (S1HL). **a** Time-frequency representation of spectral power in S1HL of naive mice (*n* = 11, left) and again 4 days after CFA injection in the contralateral hindpaw (right) during recording sessions without stimulation (grand mean, 15–25 episodes per animal). **b** Mean power in the theta (4–8 Hz), alpha (8–12 Hz), beta (15–29 Hz), gamma (30–100 Hz), low gamma (30–60 Hz) and high gamma (60–100 Hz) frequency bands for 9 s unstimulated episodes shown in **a** in the naive and CFA-induced inflammatory state (*n* = 11; *p < 0.05, two-way repeated measures ANOVA with Bonferroni multiple comparison test). **c–f** Quantification of oscillatory power in defined frequency bands for the 1 s pre-stimulation period and over 2 s post-stimulation (represented in 250 ms time bins) in the S1HL of naive and CFA-inflamed mice during all 0.6 g von Frey filament application trials irrespective of the response behavior (*n* = *7* per group in all panels; *p < 0.05 compared to naive pre-stimulation baseline, ^#^*p* < 0.05 compared to own respective pre-stimulation baseline, two-way repeated measures ANOVA with Bonferroni multiple comparisons). Data are represented as mean ± S.E.M.

We next addressed the question whether resting oscillatory rhythms in the S1HL are modulated in persistent pain states. In mice with inflammatory pain induced by unilateral injection of Complete Freund’s adjuvant (CFA) in the hindpaw, we observed that already in the basal state, i.e., absence of noxious stimulation, a significantly enhanced power of resting gamma activity was observed in the contralateral S1HL as compared to naive mice when tested over randomly selected time periods (Fig. 2a, b). Activity in the alpha frequency range was also markedly increased in inflamed mice, whereas theta and beta rhythms did not change (Fig. 2a, b). Because mechanical nociceptive thresholds drop to 0.6 g or below in inflamed mice, we tested evoked activity across naive or CFA-treated groups, taking an average of activity across all trials. We noted that overall gamma activity, but not over the alpha, theta or beta range, was consistently and significantly higher in the S1HL of inflamed mice upon stimulation with 0.6 g force than in naive and the pre-stimulation baseline (Fig. 2c–f).

### Increased gamma activity correlates with nociceptive paw withdrawal

**Fig. 3:**
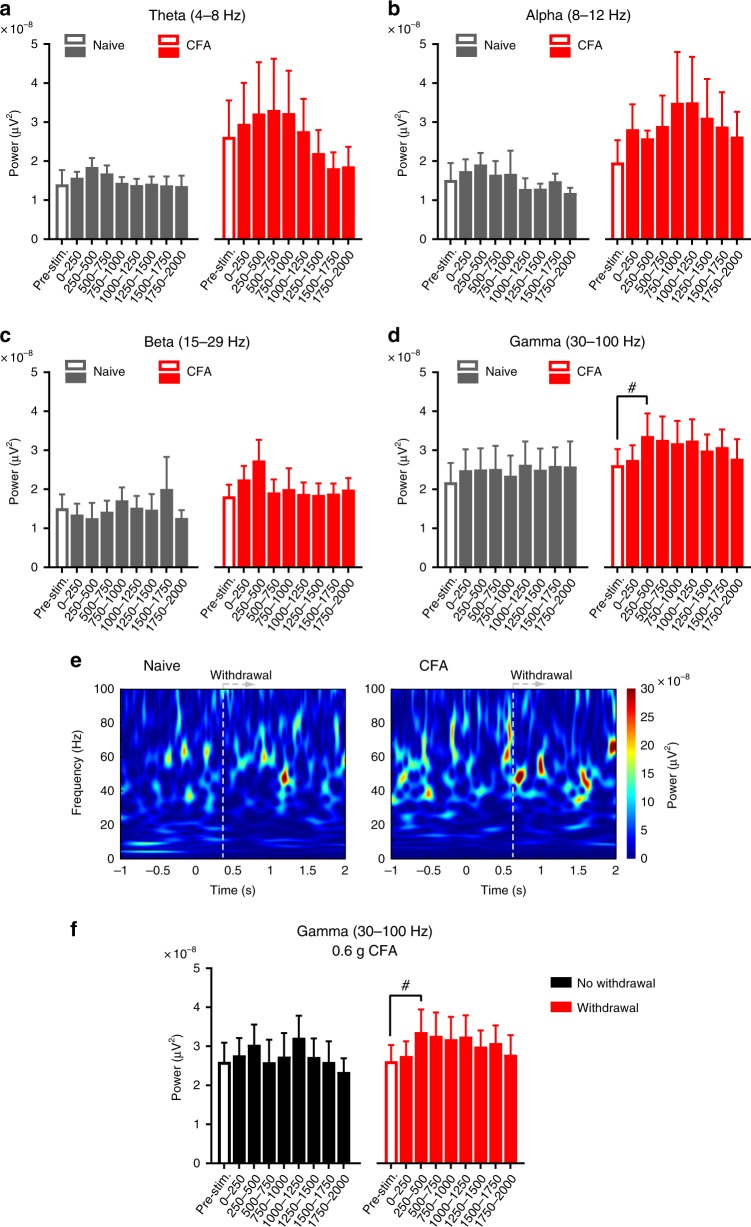
Rise in S1 gamma activity correlates with withdrawal responses during nociceptive hypersensitivity. **a–d** Quantification of oscillatory power in defined frequency bands for the 1 s pre-stimulation period and over 2 s post-stimulation (represented in 250 ms time bins) in the S1HL of naive and CFA-inflamed mice in withdrawal trials to 0.6 g von Frey filament applications (*n* = *7* per group in all panels; *p* > 0.05 compared to naive pre-stimulation baseline, ^#^*p* < 0.05 compared to own respective pre-stimulation baseline, two-way repeated measures ANOVA with Bonferroni multiple comparisons). **e** Time-frequency representations of spectral power in the S1HL of naive (left) and CFA-inflamed (right) mouse during paw withdrawal trials in response to 0.6 g filament applications (*n* = 7 mice). **f** Quantification of oscillatory power in the gamma frequency band in S1HL of CFA-inflamed mice for trials without or with a paw withdrawal evoked by 0.6 g von Frey filament application (*n* = 7; ^#^*p* < 0.05 compared to own respective pre-stimulation baseline, two-way repeated measures ANOVA with Bonferroni multiple comparisons). The data shown in **d** (red bars; CFA) are presented in **f** (red bars; CFA withdrawal). Data are represented as mean ± S.E.M.

Naive mice only occasionally showed withdrawal to 0.6 g and at this near-threshold stimulus, we did not consistently observe any potentiation of gamma or other bands of activity in withdrawal trials (Fig. 3a–e). In contrast, inflamed mice frequently demonstrated paw withdrawal to 0.6 g and not only developed an increased baseline gamma activity, but also showed a significant rise over baseline in the 250 and 500 ms intervals following von Frey application in withdrawal trials (Fig. 3d, f); this rise in gamma came about faster than withdrawal to 0.6 g in individual trials (Fig. 3e; mean withdrawal latencies of 0.46 ± 0.11 s and 0.61 ± 0.15 s in naive and inflamed mice, respectively)). Thus, a rise in S1 gamma activity correlated with nociceptive hypersensitivity, which was further confirmed when we compared withdrawal versus no-withdrawal trials for 0.6 g within the CFA group – trials eliciting withdrawal to the reduced threshold of 0.6 g were associated with a significant rise in gamma power as compared to trials in which inflamed mice did not withdraw to 0.6 g despite showing an increase in resting gamma. The power of some of the other (lower) frequency bands in response to 0.6 g stimulation showed high variability and lack of statistically significant differences (Fig. 3a–e). When suprathreshold noxious von Frey stimulation (2 g) was employed, inflamed mice showed a significant increase in power over the gamma and alpha frequency bands (Supplementary Fig. 2). Taken together, these results suggest that gamma and alpha power in the S1HL is enhanced in resting state and in response to noxious stimulation in mice with paw inflammation; however, only gamma power was enhanced in inflamed mice with stimuli that are typically non-noxious or near-threshold under naive conditions, suggestive of an electrophysiological correlate of inflammatory mechanical hypersensitivity.

### Optogenetic gamma entrainment in S1 cortex leads to hypersensitivity

**Fig. 4:**
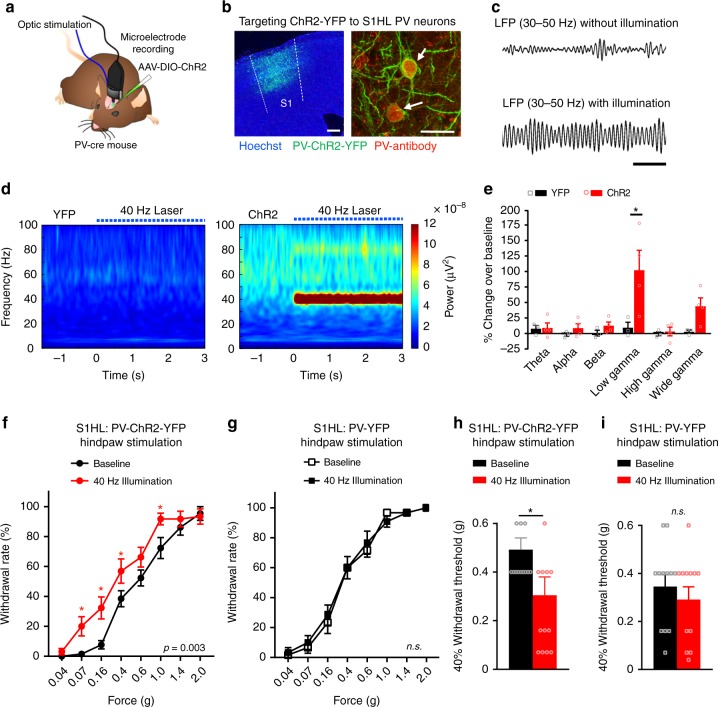
Enhancement of gamma-band oscillation power in the S1 cortex induces nociceptive hypersensitivity in mice. **a** Scheme of the experimental procedure. **b** Left: Expression of cre-dependent ChR2-EYFP in the S1HL of a *PV-cre* mouse (PV-ChR2-YFP). Scale bar represents 250 µm. Right: Co-labeling of cells stained with an anti-PV antibody and expressing ChR2-YFP in the S1HL. Scale bar represents 25 µm. **c** LFP bandpass filtered between 30 Hz and 50 Hz of a PV-ChR2-YFP mouse with and without 40 Hz laser illumination (472 nm; 10 trials). Scale bar represents 200 ms. **d** Time-frequency power representations during a 3 s illumination period with 40 Hz laser pulses (indicated by dotted blue line) of PV-ChR2-YFP mice (right) or control mice expressing YFP alone in PV neurons (left). Spectrograms represent grand means (*n* = 3 mice per group and 20 trials each). **e** Mean power changes (represented as % ERP over the 3 s 40 Hz illumination period normalized to a 1 s baseline period before illumination) in the theta (4–8 Hz), alpha (8–12 Hz), beta (15–29 Hz), low gamma (30–60 Hz), high gamma (60–100 Hz), and gamma (30–100 Hz) frequency bands in PV-YFP versus PV-ChR2-YFP animals (*n* = 3 in each group). **f**, **g** Paw withdrawal frequencies to graded von Frey stimulation of the hindpaw at baseline and during 40 Hz illumination in the contralateral S1HL of **f** PV-ChR2-YFP mice (*n* = 13; *p* = 0.003) and **g** PV-YFP control mice (*n* = 12; *p* = 0.69). **h**, **i** The 40% mechanical thresholds (from **f** and **g**) are shown at baseline and during 40 Hz illumination in **h** PV-ChR2-YFP mice (*n* = 13; *p* = 0.02) and **i** PV-YFP mice (*n* = 12; *p* = 0.42). *p < 0.05, two-way repeated measurements ANOVA with Bonferroni multiple comparisons (**e**, **f**, **g**) and Student’s paired *t*-test (**h**, **i**). *p*-values in **f** and **g** indicate significance of the effects of 40 Hz illumination treatment over the entire stimulus–response curve; *n.s*., not significant. Data are represented as mean ± S.E.M.

To test the functional relevance, we modified a model for entraining cortical gamma previously described in the S1 barrel cortex^14,15^ by optogenetically entraining strong gamma activity in the S1HL and pairing it with hindpaw stimulation (Fig. 4a). We conditionally directed the expression of the light-activable cation channel, Channelrhodopsin 2 (ChR2), tagged with yellow fluorescent protein (YFP) specifically to GABAergic PV interneurons of the S1HL (PV-ChR2-YFP) using recombinant adeno-associated virus-mediated delivery and the Cre-loxP system (Fig. 4b). Channelrhodopsin-expressing virus did not spread to the neighboring regions, including the M1 cortex and the area of optogenetic illumination was restricted and excluded the M1 area (Supplementary Fig. 3). Application of blue light pulses to stimulate ChR2 activation via optic fibers implanted in the S1HL enhanced local field potentials (LFPs) in the S1HL (Fig. 4c). As a negative control, YFP alone was similarly expressed in PV neurons in the S1HL (PV-YFP). Driving the activity of PV neurons in the S1HL at 40 Hz optogenetically evoked a strong gamma rhythm in the S1HL in awake, behaving PV-ChR2-YFP mice but not PV-YFP mice (Fig. 4d). Optogenetically-evoked 40 Hz gamma was enhanced by approximately 100% over baseline values in PV-ChR2-YFP mice (Fig. 4e). When von Frey mechanical stimuli at varying intensities were applied to the contralateral hindpaw concomitant to photo-illumination, mice with optogenetically entrained 40 Hz gamma rhythm, but not control PV-YFP mice, showed mechanical hypersensitivity as compared to their own baseline sensitivity (Fig. 4f, g). Moreover, PV-ChR2-YFP mice, but not control PV-YFP mice, showed a significant decline in nociceptive response thresholds upon entraining S1 gamma (Fig. 4h, i).

**Fig. 5:**
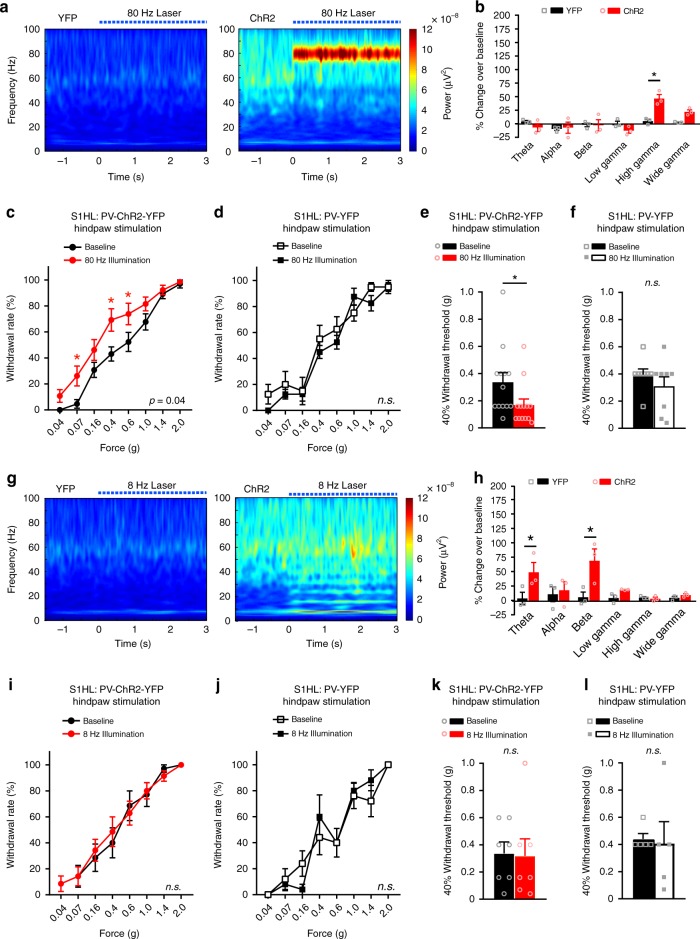
Optogenetic entrainment of high gamma, but not alpha-theta rhythms, in the S1 leads to nociceptive hypersensitivity. **a** Time-frequency power representations during a 3 s illumination with 80 Hz laser pulses (dotted blue line) of PV-ChR2-YFP mice (right) or PV-YFP mice (left). **b** Mean power changes (represented as % ERP over illumination period normalized to a 1 s baseline) in the indicated frequency bands for PV-YFP versus PV-ChR2-YFP animals (*n* = 3 in each group). **c**, **d** Paw withdrawal frequencies to graded von Frey stimulation of the hindpaw during 80 Hz illumination in the contralateral S1HL as compared to baseline measurements in **c** PV-ChR2-YFP mice (*n* = 13; *p* = 0.04) and **d** PV-YFP control mice (*n* = 8; *p* = 0.14). **e**, **f** The 40% mechanical thresholds (from **c** and **d**) at baseline and during 80 Hz illumination in **e** PV-ChR2-YFP mice (*n* = 13; *p* = 0.008) and **f** PV-YFP mice (*n* = 8; *p* = 0.8). **g** Time-frequency representation during a 3 s illumination period with 8 Hz laser pulses and the corresponding mean power changes (**h**) (as described under **a** and **b** above, respectively). In **a**, **b**, **g**, and **h**, spectrograms represent grand means (*n* = 3 mice per group and 20 trials each). **i**, **j** Paw withdrawal frequencies to hindpaw mechanical stimulation during 8 Hz illumination in the contralateral S1HL compared to baseline. **i** PV-ChR2-YFP mice (*n* = 7; *p* = 0.88) and **j** PV -YFP mice (*n* = 5; *p* = 0.09). **k**, **l** The 40% mechanical thresholds (from **i** and **j**) at baseline compared to during 8 Hz illumination in **k** PV-ChR2-YFP mice (*n* = 7; *p* = 0.82) and **l** PV-YFP mice (*n* = 5; *p* = 0.80). *p < 0.05, two-way repeated measurements ANOVA with Bonferroni multiple comparisons (**b**, **c**, **d**, **h**, **i**, **j**) and Student’s paired *t*-test (**e**, **f**, **k**, **l**). *p*-values in **c**, **d**, **i**, and **j** represent significance of the effects of illumination treatment over the entire stimulus–response curve; *n.s*., not significant. Data are represented as mean ± S.E.M.

In some studies in humans, the strongest increase in pain-associated gamma-band oscillations has been reported in the high gamma domain (i.e., 70–80 Hz)^9,28^. We, therefore, entrained high gamma activity at 80 Hz in the S1HL of PV-ChR2-YFP mice (Fig. 5a) at moderate intensity, i.e., approximately 50% over baseline values (Fig. 5b), which matched the intensity of physiologically-evoked gamma upon noxious stimulation (Fig. 1d). As with low gamma rhythms, entraining high gamma in the S1HL led to mechanical hypersensitivity, seen as a leftward shift in the stimulus–response curve and a reduction in nociceptive threshold in PV-ChR2-YFP mice, but not in control PV-YFP mice (Fig. 5c–f).

As additional controls, we optogenetically entrained an 8 Hz oscillation, which constitutes a theta or lower alpha rhythm in a comparable magnitude (Fig. 5g, h) and led to harmonic peaks in the beta region (15–29 Hz; Fig. 5h) in the S1HL. Moreover, to tease out any potential contribution of intermediate frequencies, we optogenetically entrained a 16 Hz rhythm in the S1HL (Supplementary Fig. 4). Neither 8 Hz stimulation nor 16 Hz stimulation led to any significant deviations in mechanical nociceptive sensitivity in either PV-ChR2-YFP mice or PV-YFP mice (Fig. 5i–l and Supplementary Fig. 4a–d). Thus, gamma activity, but not other oscillatory rhythms in the frequency bands between 8–29 Hz, was associated with nociceptive hypersensitivity.

### Gamma increases during nociceptive responses are not motor-related

**Fig. 6:**
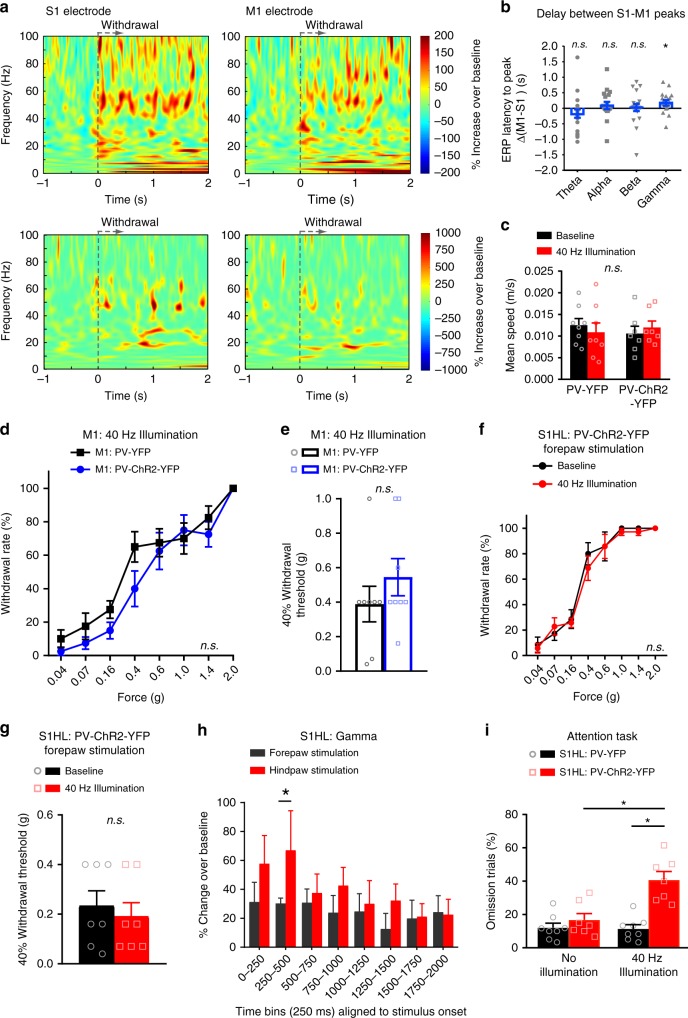
Enhanced gamma activity evoked by noxious stimulation is not related to motor activity. **a** Averaged (top row, *n* = 4 mice) and single trial examples (bottom row) of time-frequency representation of spectral modulation recorded simultaneously in S1HL (left) and M1 (right) of a paw withdrawal trial in response to 2 g von Frey stimulation. Power is coded as % ERP representing the deviation from the mean over a 1000 ms baseline period. **b** Comparison of difference in the peak time of stimulation-evoked ERP across frequency bands between M1 and S1 electrodes in individual trials (*n* = 20 withdrawal trials from four mice). **c** Mean running speed (analyzed over 20 min) of PV-YFP (*n* = 8) and PV-ChR2-YFP (*n* = 7) mice at baseline or S1HL 40 Hz illumination (*p* > 0.05, two-way ANOVA). **d** Paw withdrawal frequencies to hindpaw mechanical stimulation during 40 Hz illumination of the contralateral motor cortex (M1) in mice expressing either YFP or ChR2-YFP in M1 (*n* = 8/group; *p* = 0.30) and **e** the corresponding 40% mechanical thresholds (*p* = 0.31). **f** Withdrawal frequencies to forepaw von Frey stimulation at baseline and 40 Hz illumination of the S1HL cortex of mice expressing PV-ChR2-YFP in the S1HL (*n* = 7; *p* = 0.94) and **g** the corresponding 40% mechanical thresholds (*n* = 7; *p* = 0.69). **h** Comparison of change in mean event-related perturbation in S1HL power over a 1000 ms baseline in the high gamma frequency band evoked by stimulating either the contralateral forepaw or hindpaw with a 2 g von Frey filament (5–7 applications per paw) (*n* = 7; *p < 0.05). **i** The percentage of omission of attention trials in the 5-choice serial reaction task in the absence and presence of 40 Hz illumination of mice expressing PV-YFP (*n* = 8) or PV-ChR2-YFP (*n* = 7) in the S1HL (*p* < 0.0001). *p < 0.05, one-sample *t*-test for deviation from zero (**b**), two-way repeated measures ANOVA with Bonferroni multiple comparisons (**d**, **f**, **h**, **i**), unpaired *t*-test (**e**), Student’s paired *t*-test (**g**). *n.s*., not significant. Data are represented as mean ± S.E.M.

We performed a series of control experiments to rule out that the change in gamma activity evoked by noxious stimulation is related to motor activity. First, in dual site recordings in the S1HL and the M1 upon mechanical noxious hindpaw stimulation (Fig. 6a), we observed that activity peaked first in the S1 and then in the M1, as seen via a significant elevation over zero values upon calculating the delta of latencies in the M1 versus the S1HL (Fig. 6b). In contrast, with respect to theta, alpha and beta frequency bands, there was no consistency as to whether activity originated earlier in the S1 or the M1 (Fig. 6b). This suggests that noxious stimulus-evoked increase in gamma band oscillation (GBO), which we observed in the S1HL, is not a result of preceding GBO activity in the M1, but rather vice versa. Secondly, the area of virus injection in the S1HL as well as the optogenetic illumination excluded the M1 cortex (Supplementary Fig. 3) and there was no change in gait or speed of locomotion in mice with optogenetic entraining of GBO in the S1HL (Fig. 6c). Finally, optogenetically entraining a 40 Hz gamma rhythm in the M1 did not lead to any mechanical hypersensitivity in PV-ChR2-YFP mice, unlike the outcome of entraining GBO in the S1HL, but rather elicited a trend for mechanical hypoalgesia as compared to control PV-YFP-expressing mice (Fig. 6d, e). These results suggest that motor activity contributed neither to GBO in S1 nor to the hypersensitivity observed upon entraining GBO in the S1.

### Gamma activity increase in S1 cortex is not associated with salience

Another alternative explanation for the nociceptive hindlimb hypersensitivity we observed upon entraining GBOs in the S1HL is that gamma activity leads to an overall increase in attention or alertness, leading to an increased salience in perception of sensory stimuli. We undertook three experiments to address this possibility. One, we entrained 40 Hz gamma activity in the S1HL in PV-ChR2-YFP mice and applied mechanical stimuli over the non-noxious and noxious range to the forepaw instead of the hindpaw. Enhanced gamma activity in the S1HL did not lead to change in mechanical sensitivity or mechanical thresholds of the forepaw (Fig. 6f, g), speaking against a generalized increase in attention. Second, noxious stimulation to the hindpaw, but not to the forepaw, led to a significant increase in gamma activity in the S1HL (Fig. 6h). Finally, employing a widely accepted classical behavioral paradigm of attention in mice, namely the 5-choice serial reaction time (5-CSRT) task^29^, which is used commonly to assess visuospatial attention and motor performance in rodents, we noted that PV-ChR2-YFP mice with gamma entrainment in the S1HL showed a marked increase in omission rates, i.e., a significant decrease in attention performance, compared to control PV-YFP mice lacking gamma entrainment (Fig. 6i). Thus, nociceptive hypersensitivity evoked by gamma entrainment in the S1HL is not associated with enhanced attention.

### Gamma entrainment in S1 cortex induces negative affect and aversion

**Fig. 7:**
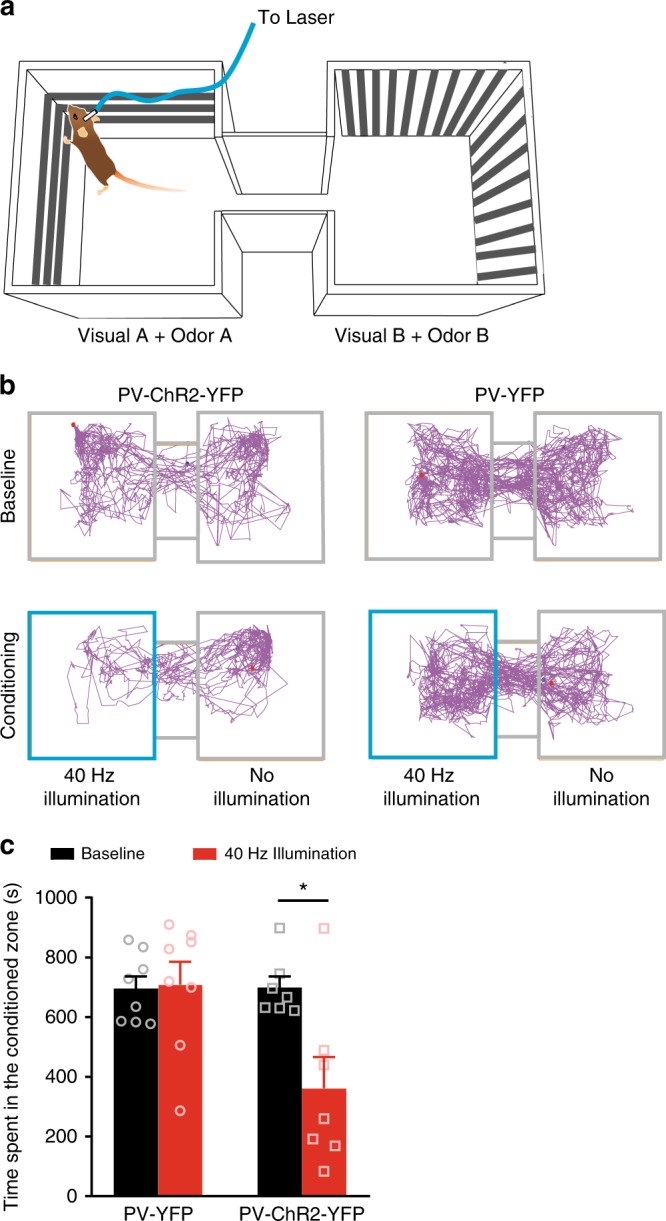
Strong gamma activity in the S1 cortex induces real-time conditioned place avoidance (CPA). **a** Scheme of the experimental procedure for testing CPA to gamma rhythms induced in the S1HL. **b** Example of tracking plots from a PV-YFP and PV-ChR2-YFP mouse recorded at baseline and during conditioning with 40 Hz illumination in the S1 cortex; area highlighted in blue indicates the chamber paired with optogenetic gamma induction in the S1 cortex. **c** Quantification of the total time spent in the gamma-conditioned chamber at baseline and during conditioning (40 Hz illumination in the S1 cortex) of mice expressing either PV-YFP alone (*n* = 8) or PV-ChR2-YFP (*n* = 7) in the S1 cortex. *p < 0.05, two-way repeated measures ANOVA with Bonferroni multiple comparisons. Data are represented as mean ± S.E.M.

It is currently difficult to unequivocally study negative affect in rodents. In the field, preference or avoidance paradigms are currently employed to address aversive states^30^. We, therefore, devised a real-time conditioning paradigm, in which mice learned to associate specific contextual cues, such as visual patterns and odor cues, with gamma activity in the S1 cortex (Schematic in Fig. 7a; tracking plot examples of individual mice in Fig. 7b and quantitative summary in Fig. 7c). During the real-time conditioning phase, 40 Hz gamma activity was optogenetically induced in the S1 of PV-ChR2-YFP mice in association with a particular chamber with specific contextual cues, while 40 Hz laser stimulation in PV-YFP mice served as a control. PV-ChR2-YFP mice spent significantly less time in the chamber linked with 40 Hz gamma activity, demonstrating a conditioned aversion for the place in which animals were subjected to enhanced S1 gamma activity (Fig. 7b, c). In contrast, control PV-YFP mice spent equal time in the conditioned chamber as baseline (Fig. 7b, c). Thus, optogenetically strengthening the power of GBOs in the S1 cortex elicited aversive avoidance in mice.

### Pain modulatory centers recruited by entraining S1 gamma activity

**Fig. 8:**
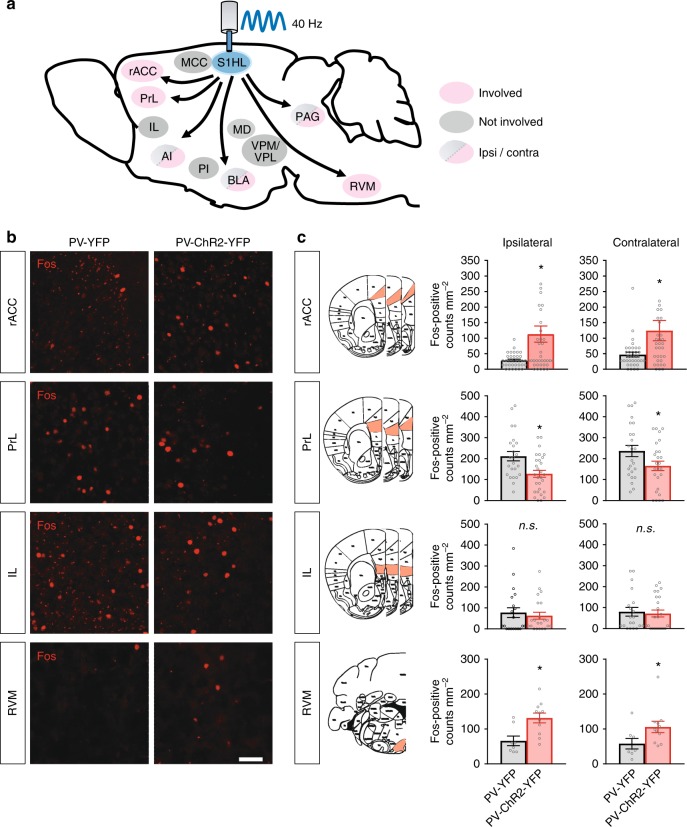
Functional c-Fos mapping (for neuronal activation) of brain regions altered in activity following optogenetically induced gamma activity in the S1HL cortex in PV-ChR2-YFP mice. **a** Schematic representation of pain-related brain areas that were analyzed in the experiment. Red shading: regions altered in activity; gray shading: regions not altered. Arrows do not necessarily indicate direct afferent connections. Regions showing differences in Fos expression between the ipsi- versus contralateral areas are additionally indicated with the corresponding color codes in the scheme. **b** Typical example of changes in Fos expression in the rACC, the RVM, PrL, and IL. **c** Quantitative summary of changes in c-Fos expression in diverse brain regions following entrainment of gamma rhythm in the S1HL in the PV-ChR2-YFP mice (*n* = 7–9 mice per brain region analyzed) or blue photo-illumination of the S1HL in control PV-YFP mice (*n* = 6–8 mice per brain region analyzed). Differences in Fos-positive counts between YFP and ChR2-YFP mice following 40 Hz illumination were analyzed in the ipsilateral and contralateral rostral anterior cingulate cortex (rACC; *p* = 0.003 and 0.07, respectively), prelimbic cortex (PrL; *p* = 0.003 and 0.047, respectively), infralimbic cortex (IL; *p* = 0.60 and 0.75, respectively) and the rostroventral medulla (RVM; *p* = 0.005 and 0.049, respectively). The RVM analyzed included the raphe magnus nucleus and gigantocellular reticular nucleus, alpha region. *p < 0.05, unpaired *t*-test; *n.s*., not significant. Scale bars represent 50 µm in **b**. Data are represented as mean ± S.E.M.

Next, with a view towards understanding mechanisms, we aimed to unravel the nature of brain networks that are activated downstream of S1 gamma activity. As a surrogate for activity, we analyzed expression patterns of the activity-induced immediate early gene product, Fos^31^. A schematic overview of pain-related brain regions tested for changes in Fos expression upon S1 gamma induction is given in Fig. 8a, examples of individually immunostained regions are shown in Fig. 8b and Supplementary Fig. 5a. Quantitative summary of Fos-positive neurons is given in Fig. 8c and Supplementary Fig. 5b. The rostral anterior cingulate cortex (rACC), which is implicated in pain affect^32^, showed a very large increase in the number of Fos-positive cells directly after inducing 40 Hz GBOs in PV-ChR2-YFP mice as compared to control mice (Fig. 8b, c). Interestingly, the the prelimbic cortex (PrL) domain of the prefrontal cortex (PFC), which is implicated in pain^3,33^, showed a significant decrease in the number of Fos-positive cells in PV-ChR2-YFP mice as compared to control mice upon 40 Hz illumination (Fig. 8b, c). In contrast, several cortical areas, such as the mid-cingulate cortex (MCC) area, the infralimbic cortex (IL), and the posterior insula (PI), as well as diverse nociception-related thalamic nuclei did not show significant changes (Fig. 8b, c and Supplementary Fig. 5). The anterior insula (AI), the basolateral amygdala (BLA) and the periaqueductal gray (PAG) showed significantly increased number of Fos-positive cells only in the hemisphere contralateral to 40 Hz illumination in PV-ChR2-YFP mice as compared to control PV-YFP mice (Supplementary Fig. 5). Notably, gamma activity in the S1 led to a marked increase in the activity of neurons in the midbrain nuclei located in the rostroventral medulla (RVM) that are prominently involved in descending modulation of pain. Particularly, the nucleus raphe magnus (RMg) and the nucleus reticularis gigantocellularis pars alpha area (GiA) together showed a significant increase in the number of Fos-positive neurons in response to S1 gamma (Fig. 8a–c).

### Functional contribution of descending serotonergic pathways

**Fig. 9:**
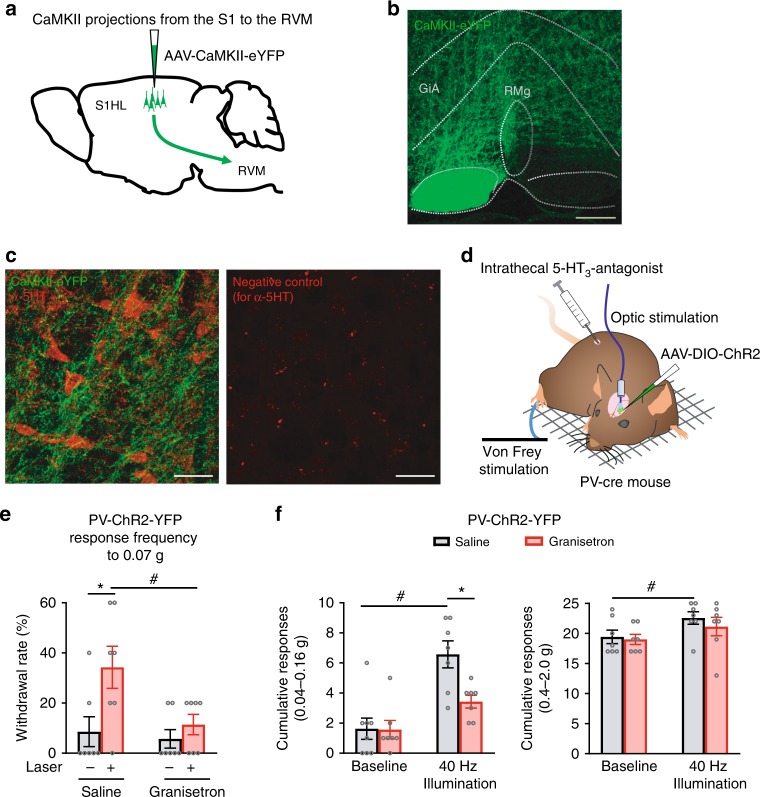
Traced projections from the S1HL to important pain modulatory centers in the rostroventral medulla (RVM) and functional contribution of descending serotonergic pathways to nociception by gamma activity in the S1. **a** Schematic representation of the viral tracing experiments. **b** Example image of viral tracing of projections from pyramidal neurons expressing eYFP (in green) in the S1HL to the RVM (observed in the raphe magnus nucleus (RMg) and the gigantocellular reticular nucleus, alpha (GiA)). **c** Immunohistochemical identification of serotonergic neurons (in red) in the proximity of afferent projections from the S1HL to the RMg. The right panel depicts an example of a negative control image for anti-5HT staining. **d** Scheme of the experimental procedure for **e** and **f** is shown. **e** Suppression of hypersensitivity induced by optogenetically enhancing gamma activity in the S1HL upon intrathecal injection of the serotonin receptor antagonist, granisetron (*n* = 7), but not vehicle (saline, *n* = 8). **f** The cumulative responses to lower (left panel) and higher intensities (right panel) of von Frey forces applied to the paw. ^#^*p* < 0.05 compared to respective baseline, *p < 0.05 compared between groups, two-way ANOVA with Bonferroni multiple comparisons. Scale bars represent 200 µm and 50 µm in **b** and **c**, respectively. Data are represented as mean ± S.E.M.

In particular, the RMg and GiA contain the cell bodies of serotonergic neurons, which mediate descending facilitation of spinal nociceptive processing^34,35^. We, therefore, performed tracing of connectivity of excitatory neurons, which are the output neurons that are modulated by local PV interneurons, in the S1 to the RVM. Upon injection of AAV-virions expressing GFP specifically in the S1HL cortex, GFP-expressing afferent projections were readily detected in the RMg and the GiA (schematic in Fig. 9a and examples in Fig. 9b). Several axonal varicosities of S1 projections were observed in close proximity of serotonergic neurons in co-immunohistochemistry experiments (Fig. 9c). In a second set of experiments involving pharmacological manipulations and nociceptive behavior (schematic in Fig. 9d), we found a direct functional significance for serotonergic modulation by cortical gamma activity. We induced 40 Hz gamma oscillatory activity by optogenetic stimulation of PV neurons in the S1, as described above in Fig. 4, in mice that were intrathecally injected with either vehicle or the drug granisetron, a blocker of descending serotonergic facilitation^36^, in the spinal cord. As shown in Fig. 4, optogenetically entraining a 40 Hz gamma rhythm in the S1 induced mechanical allodynia in vehicle (saline)-treated mice, which was abrogated upon granisetron-induced blockade of descending serotonergic function (Fig. 9e, f). Responses to 0.07 g as a typically non-noxious intensity of mechanical stimulation are shown in Fig. 9e and cumulative responses to all low force stimulation intensities are shown in the left panel in Fig. 9f. At higher intensities of mechanical stimulation, the mild but significant enhancement of sensitivity elicited by 40 Hz gamma activity in the S1 also did not occur in spinal granisetron-treated mice (Fig. 9f, right panel). Taken together, these morphological and functional experiments suggest a strong downstream contribution of descending serotonergic facilitation in the pronociceptive functions of gamma activity in the S1 cortex, particularly so with respect to allodynia rather than hyperalgesia.

## Discussion

Here, we report a mouse model revealing the functional significance of pain-related gamma-band activity in the S1 cortex and provide an elucidation of functional circuits involved in modulation of nociception and pain by gamma oscillations in S1.

Previous studies involving transcranial recordings and electrocorticogram measurements have reported changes in electrical activity over wide frequency ranges in rodents upon application of noxious stimuli, such as laser stimulation^12,37^. In direct intracortical recordings, we observed that although the gamma power in S1 always increased with a near-threshold mechanical stimulus, the increase was significantly higher when that particular stimulus was noxious and elicited nocifensive behaviors. This is consistent with observations in human subjects that although non-nociceptive somatosensory stimulation can also elicit gamma activity in the S1 cortex (e.g. ref. ^38^), gamma strength increases to a significantly stronger level in humans around the pain threshold when the laser stimulus was perceived to be noxious as compared to when it was not^9^.

One potential and highly important caveat, which we clarified in detail, is whether the noxious stimulus-induced increase in gamma oscillatory activity in the S1 involved the M1 cortex or resulted from increased motor activity associated with paw withdrawal. Several converging lines of evidence argue against it. First, noxious stimulus-evoked gamma activity increased rapidly in the S1 cortex within the first 250 ms upon stimulus application, i.e., prior to paw withdrawal; second, in mice with ongoing inflammatory pain, basal gamma strength increased although no-withdrawal responses were evoked; third, entraining S1 GBOs did not improve motor function; fourth, directly entraining GBOs in the motor cortex led to a trend of hypoalgesic behavior, opposite to the hyperalgesia seen with S1 gamma, our data are thus consistent with studies in humans reporting the utility of gamma stimulation in the motor cortex in inducing analgesia and pain relief^39^, and harmonize well with studies, which show that transcranial direct current stimulation or repetitive transcranial magnetic stimulation over the motor cortex reduces the perception of painful stimuli via engaging descending modulatory projections^40,41^; fifth, occasional paw withdrawal to low intensity stimuli was not associated with enhancement of S1 gamma, indicating an uncoupling of the act of paw withdrawal from gamma enhancement; sixth, in functional experiments, modulating gamma power, not only influenced withdrawal responses but also aversion-related voluntary behaviors that do not involve stereotyped or reflexive motor functions. This is consistent with increased gamma power in humans that is associated with pain perception in the absence of an overt protective motor response.

An increase in S1 theta power has been reported in rats upon application of a chemical noxious stimulus, capsaicin^42^. Here, we did not find any significant alteration in theta, beta, and alpha activity in the S1 between trials involving a response to a mechanical noxious stimulus and trials that did not. Moreover, behavioral mechanical hypersensitivity to typically innocuous or near-threshold stimuli in inflamed mice did not correlate with any rhythms apart from GBOs and simulating activity in the theta-alpha range or the beta range had no impact on mechanical nociception. It is possible that theta, beta, and alpha rhythms in the S1 are functionally associated with aspects other than nociception or objective or subjective aspects of pain perception.

In contrast, our data revealed a causal link between the enhancement of S1 GBOs and mechanical hyperalgesia as well as allodynia, suggesting that increased gamma strength in the cortex primes a state of readiness to protect from a potentially damaging stimulus by engaging descending facilitation, which is reminiscent of the state of hypersensitivity associated with pathological pain states. Consistent with this notion, we observed a higher basal gamma rhythm in animals with inflammatory pain. This may have important implications for changes in pain perception in brain disorders that impact on the strength of cortical gamma and function of parvalbumin interneurons, such as schizophrenia^43,44^. However, we also acknowledge the alternative possibility that the physiological or optogenetic enhancement of GBOs in the S1 cortex changes the quality of the elicited percept, rendering it unnatural or abnormal, independently of its ability to induce pain. This may link GBO entrainment to non-painful paresthesias that could also account for behavioral hypersensitivity. Nevertheless, two observations are noteworthy. First, we only observed an increase in S1 gamma in inflamed mice to mechanical stimuli that are normally sub-threshold, thus matching the behavioral correlate of allodynia. Second, we also observed that S1 gamma entrainment elicited tonic aversive behaviors, suggesting that the abnormal percept, if generated, is unpalatable or unpleasant and thereby linked to pain. Overall, these observations argue for the utility of rodent models in testing a mechanistic framework for S1 gamma activity in nociception, aversion and paresthesia, and studying alterations thereof in pathological states.

In humans and primates, prefrontal gamma activity has also been discussed to amplify attention or vigilance^4,45^, and cortical gamma has been suggested to contribute to attentional effects on pain-related behaviors^6^. However, we observed that inducing gamma activity selectively in the hindlimb representation area of S1 did not bring about increased responsivity to noxious stimuli applied to other dermatomes in the body, such as the forelimb, speaking against an overall heightened state of arousal or vigilance in these animals. Analyses involving gamma entrainment in the S1 cortex during attention-related tasks in a widely accepted behavioral test also negated the notion that enhancing or entraining S1 gamma leads to an overall enhancement of attention or vigilance. Moreover, in a study on fibromyalgia syndrome patients with perceived hypervigilance, no increase in cortical gamma activity was found^46^. Furthermore, studies on human subjects also suggest that low frequency oscillations, not gamma activity, in the S1 correlate with unspecific stimulus-triggered attentional processing (saliency) of the applied sensory stimulus^47^. Taken together with the above, our observations suggest that gamma oscillations in S1 induce nociceptive hypersensitivity independently of attentional factors.

Our findings link S1 gamma activity to voluntary avoidance behaviors and negative affect, which may or not be pain-related negative affect. This is noteworthy because in lesioning or silencing studies in rodents, the rACC, but not the S1 cortex, has been linked to aversive behavior^30,32^. It should also be noted, however, that the concept of strict dichotomy between the lateral somatosensory pathway, involving the S1 in the sensory component of pain and the medial pathway, involving the cingulate cortex, in the affective component of pain, has been increasingly challenged over the recent years^48,49^. We did, however, observe that high power gamma activity in the S1 was associated with increased number of Fos-positive cells in the rACC, but not the MCC (which is not involved in pain affect modulation^49^), supporting a potential contribution of the rACC. Indeed, gamma oscillatory activity has been suggested to facilitate information flow across wide networks and enhance network coherence^50^, and thereby the strength of gamma-band activity in S1 evoked by noxious stimuli may represent a threshold for coupling with networks involved in pain affect. Alternatively, gamma oscillatory activity in the S1 may constitute a neural substrate of short-term regulation at early stages of pain processing via top–down modulation from other centers in the pain network, as hypothesized in a study on humans^28^. Interestingly, a recent study in human subjects tested cortical gamma activity during bottom–up and top–down modulation of acute pain and concluded that the current context is most important in shaping the role of gamma activity in pain^51^.

Importantly, in Fos-based activity mapping analyses, we found evidence for both direct afferent connections to as well as Fos-based activity changes in the RVM nuclei involved in descending facilitation of nociception. This, combined with the functional outcome of our pharmacological manipulations and nociceptive behavior, indicates that mechanical allodynia induced by gamma activity in the S1 involves descending serotonergic facilitation and thus supports our view that pain facilitation by S1 gamma may be independent of attentional networks.

Of note, regions that have been directly linked to nociceptive hypersensitivity, such as the MCC^49^, or the PI, which has been suggested to work as a ‘how much’ detector in pain states^52^, were not significantly affected by increased gamma power in the S1. Furthermore, despite the significance of mutual feedforward and feedback modulation between the S1 and the thalamus in nociceptive processing^53,54^, we did not observe obvious changes in thalamic nuclei. Consistent with a previous study reporting reduced activation of the medial domain of the prefrontal cortex in rats with chronic pain^55^, we observed that Fos-positive neurons decreased below baseline in the PrL upon strengthening the power of gamma in the S1, although there are no direct afferent projections reported from the S1 to the PrL. In humans, gamma activity in the PrL was reported to encode intensity of tonic pain^56^. Whether and how increases in gamma activity in the S1 and in the prefrontal cortex upon noxious stimulation are related to each other remains unknown.

Finally, the unexpected role of cortical GABAergic interneurons in pain facilitation deserves some discussion. GABAergic interneurons are the main determinants of inhibition in the cortex^18^, and activation of GABAergic interneurons or upregulation of GABAergic markers is frequently interpreted to indicate enhanced inhibition in bioimaging and electrophysiological studies. Instead, we observed that synchronous activity of fast-spiking GABAergic interneurons induces behavioral nociceptive plasticity and facilitates nociceptive processing. Synchronously firing PV neurons can actually increase the precision and coordination of action potentials in excitatory neurons, thereby resulting in amplification and sub-threshold and suprathreshold net oscillatory activity^17,57^. PV neurons receive direct inputs from the thalamus^18^, a main relay station in the somatosensory spinothalamic pathway, and are thus ideally placed to aid the translation of incoming nociceptive signals into a local gamma rhythm in the S1. Once generated in the S1, such a gamma rhythm may facilitate the spread of activity between brain regions, and thus enhance pain perception and processing in a large network of cortical and subcortical structures^1,33^.

It must be acknowledged that several questions remain open. Since only mechanical nociception was studied here, it remains to be determined whether other modalities of nociception and pain, such as thermal or chemical, are modulated by GBOs. Secondly, this study exclusively studied GBOs in the S1 cortex, while in recent studies on human subjects, noxious stimuli also elicited enhancement of GBOs in the prefrontal^58,59^ and insula^60^ cortices. Therefore, testing this mouse model for changes in GBOs in additional brain areas will be rewarding. Finally, resolving the functional implications of S1 GBOs in enhanced pain perception versus non-painful paresthesias remains a challenge.

In summary, the results of this study indicate important functional contributions of cortical gamma oscillatory activity towards modulation of both sensory and aversive functions, which are important domains of the multidimensional experience of pain. Our results identify underlying circuits to prefrontal areas as well as medullary centers and indicate that enhanced gamma oscillatory drive can underlie hypersensitivity in persistent pain states, such as inflammatory pain. These causal and mechanistic insights hold particular significance in the light of the proposed utility of cortical gamma activity in the diagnoses as well as in treatment, e.g., in form of neurofeedback, of pain disorders^6^.

## Methods

### Animals

Experiments were performed in male and female 4- to 8-month-old *PV-Cre* mice (from three or more different litters) with a C57BL/6 background that were previously described^61^. Animals were housed with food and water ad libitum on a 12 h light/12 h dark cycle. All experimental procedures were performed according to the ethical guidelines set by the local governing body (Regierungspräsidium Karlsruhe, Germany; approval numbers 35–9185.81/G119/14 and 35–9185.81/G184/18).

### Surgical procedures

Mice were deeply anaesthetized by intraperitoneal injection of fentanyl (0.05 mg ml^−1^), medetomidine hydrochloride (1 mg ml^−1^) and midazolam (5 mg ml^−1^) mixture (4:6:16, 0.7 µl per gram body weight). Lidocaine (10%) was applied to the skin surface and a small hole was drilled above the region of interest. In vivo delivery of recombinant adeno-associated virus-mediated delivery (rAAVs) was performed by stereotactic injections. The coordinates used relative to Bregma were as follows: S1HL (posterior 0.13 mm, lateral 1.85 mm, 0.45 mm depth); M1 hindlimb region (anterior 0.3 mm, lateral 1.4 mm, 0.5 mm depth). rAVV injections of 0.5–0.8 µl was delivered over 30 min. The mice were randomly allocated to receive virus encoding rAAV5-EF1a-DIO-hChR2(H134R)-EYFP, rAAV5-EF1a-DIO-hChR2(E456T/T159C)-EYFP, rAAV5-Ef1a-DIO-EYFP, or rAAV5-CaMKIIa-EYFP (purchased from University of North Carolina Vector Core, USA). Animals were kept for 4 weeks to achieve optimal in vivo viral expression prior to experiments.

For behavioral experiments, a chronic optical fiber implant (105–230 µm in diameter, numerical aperture (NA) of 0.22) was inserted 50 µm above the viral injection site and secured on the skull with dental cement and a screw.

For electrophysiological experiments, a small craniotomy was performed and the dura mater was removed. Three animals were implanted with a 4-shank 16-channel silicon probe (A4x1-tet-3mm-150–121, mounted on a d-drive, Neuronexus), with the anterior-most shank targeting the rostral S1HL (0.1 mm anterior to Bregma, 1.9 mm lateral, 0.3 mm depth) and the posterior-most shank targeting the central S1HL (0.3 mm posterior to Bregma, 1.7 mm lateral, 0.3 mm depth). All other animals were implanted with a versadrive 4 (Neuralynx) consisting of four independently movable tetrodes arranged in a square and separated by 0.6 mm, with the anterio-lateral tetrode targeting the rostral S1HL (0.1 mm posterior to Bregma, 1.8 mm lateral, 0.3 mm depth), the anterio-medial tetrode targeting M1 (0.1 mm posterior to Bregma, 1.2 mm lateral, 0.3 mm depth), and the posterio-lateral tetrode targeting the caudal S1HL (0.7 mm posterior to Bregma, 1.8 mm lateral, 0.3 mm depth). For the combined optogenetic stimulation and electrophysiological recording experiments, animals first received an rAAV injection in S1HL and were then implanted with a versadrive 4 equipped with a custom-added optic fiber (105 µm in diameter, NA = 0.22) positioned in the center, and the four tetrodes arranged around it in the square formation described above. The stripped optic fiber was lowered to 50 µm above the site of injection and remained in place till the end of the experiment. Two stainless steel screws above the cerebellum and ipsilateral parietal cortex served as ground and reference screws, respectively. The tetrodes were implanted at a depth of 0.3 mm and the microdrive secured on the scull with dental cement. Tetrodes were made of 12 μm diameter tungsten wires (H-Formvar insulation with Butyral bond coat; California Fine Wire) and electrode tips gold-plated to an impedance of 250 kΩ (nanoZ, Neuralynx) on the day of implantation. Tetrodes were lowered to a depth of 0.6 mm during habituation to the test equipment 4–7 days before the first recording session.

Inflammation of the paw was induced by subcutaneous plantar injection of 20 μl Complete Freund’s Adjuvant (CFA, Sigma-Aldrich) under brief isoflurane anesthesia.

### Electrophysiology

Neural signals were acquired via a HS-18-MM headstage using Digital Lynx 4SX system and Cheetah data acquisition software (Neuralynx). Signals were digitized at 32 kHz and bandpass filtered between 0.1 Hz and 9000 Hz. A custom built Piezo transducer (Piezo-ceramic element, part #717770, Conrad) was used to generate an analog stimulus signal during von Frey application. This signal was bandpass filtered between 0.1 and 2000 Hz and recorded simultaneously with all other tetrode channels. Von Frey filaments (0.6 g and 2 g) were prepared to rest on a small socket of dental cement with a base of 4–5 mm in diameter and interchangeably mounted with adhesive tape on the pressure-sensing Piezo transducer. A recording session was typically divided into blocks of graded von Frey filament stimulation, with each filament applied to the plantar surface of the contralateral hindpaw 5–7 times at a minimal interval of 60 s. Paw withdrawal and no-withdrawal events were marked with a TTL signal via a pulse generator (Master-9, AMPI, Israel) connected to an external input line of the Neuralynx system following each filament application. The mechanical stimulus was applied for at least 1.5–2 s in case the paw was not withdrawn. In addition, an unstimulated block of continuous recording over a 10 min period was included in each recording session and animals in the naive baseline state also received an additional block of pin-prick stimulation at the end of the recording session, in which 5–7 pin-prick stimuli were applied to the contralateral hindpaw with a shortened insect pin glued to a 1 g von Frey filament, and mounted on the piezo transducer as described above.

### Analysis of electrophysiology data

Data analysis was performed with Brainstorm^62^, which is documented and freely available for download online under the GNU general public license (http://neuroimage.usc.edu/brainstorm) and custom Matlab scripts (The Mathworks Inc.MA, USA). Based on two-photon in vivo imaging of functional responses to mechanical stimulation of either the hind or forepaw^63^, we focused our analysis on the anterior segment of the S1HL region represented by the tetrode located 0.05–0.1 mm caudal to bregma. The anterio-medial tetrode situated in M1 in four animals implanted with a VersaDrive was used for the analysis of the temporal relationship between S1HL and M1 LFPs. All four tetrodes surrounding the optical fiber were used in assessing optogenetic effects on LFP power. As the spatial spread of the LFP extends beyond the area occupied by a single tetrode^64^, only one channel per tetrode was selected for further analysis.

The LFP signal was resampled at 1600 Hz and a bandpass filter between 0.3 and 100 Hz as well as a notch filter at 50 Hz applied. Event marks for stimulus onset and withdrawal onset were set based on the initial deflection times of the Piezo transducer signal. For von Frey stimulation analysis, recording epochs were extracted using a window ranging from 4 s before to 5 s after stimulus onset. For unstimulated recordings twenty 10-s-epochs per block were extracted for further analysis. Individual episodes were inspected visually and rejected if they contained spontaneous potential fluctuations > ± 1 mV during the assessed period to avoid artefacts.

For the analysis of time-frequency (TFR) data, the TFR power of each epoch was obtained using Morlet wavelets. The Morlet wave was designed with a central frequency of 1 Hz and a time resolution of 5 s. The resulting power output values for a frequency range from 1 to 100 Hz were multiplied by frequency to compensate for spectral flattening. An event-related perturbation (ERP) of these TFRs was calculated for von Frey stimulation and laser illumination trials from a 1000 ms baseline for every epoch. The ERP gives the deviation from the mean over baseline in %. For group analysis TFR power or ERP maps of all trials for a given stimulus–response condition were averaged for each animal. For quantification means of the ERP % or TFR power values were calculated for different time bins and frequency bands; 4–8 Hz (theta), 8–12 Hz (alpha), 15–29 Hz (beta), 30–60 Hz (low gamma), 60–100 Hz (high gamma), and 30–100 Hz (gamma) for each subject^59^. For the LFP analysis of the optical stimulation experiments the TFR power and ERP % values of a single channel from each tetrode were averaged for each animal. Spectrogram plots of either the grand average or a representative single trial were generated in Matlab using the heatmap function and jet colormap. The unstimulated spectrogram plots (Fig. 2a) were generated with the Matlab contour function using the jet colormap.

Median withdrawal latencies (Fig. 1f) for each filament strength and subject were used for the group analysis, as the distribution of withdrawal latencies were skewed. TFR analysis in respect to withdrawal onset (Fig. 1g) were performed by aligning withdrawal trials at the respective withdrawal onset, and individual no-withdrawal trials to the median withdrawal latency for a given filament strength and subject. The TFR power for each of these trials was still normalized to the 1000 ms pre-stimulus baseline period but the ERP % for the gamma-band was now calculated for 250 ms time bins with respect to withdrawal onset.

For the comparison of peak latencies between S1HL and M1 electrodes (Fig. 6a, b), simultaneous recordings from electrodes in both of these brain regions in four animals implanted with a VersaDrive were analyzed. ERP values of individual withdrawal trials resulting from 2 g filament applications in naïve animals were averaged in 50 ms bins within each of the four frequency bands. Latencies to peak for each episode and frequency band were then specified as center of the time bin of the maximum ERP % value from stimulus onset to 0.5 s after withdrawal onset. The time lag between the two electrodes was calculated for each trial and frequency band from the difference between the ERP peak latencies (M1 peak latency – S1 peak latency).

### Optical stimulation

Mice were briefly anaesthetized and optical fiber implants were attached to optical patch cables (Thorlabs GmbH, Germany) coupled to a 473 nm laser (Shanghai Laser & Optics Century Co. Ltd, China). Light pulses of 1 ms duration were applied at an intensity of 30 mW mm^−2^ at the following frequencies: 8, 16, 40, and 80 Hz, as described by Cardin et al.^14^. Light pulses were generated by a pulse generator (33220A, Meilhaus Electronic GmbH, Germany) and were applied either repeatedly for a duration of 3 s every 20 s for electrophysiological assessment, or continuously throughout a behavioral testing session.

### Behavioral tests

All behavioral tests were carried out during the light cycle of the animals. Animals underwent two 30 min acclimatization sessions in the setups used for mechanical testing. Mechanical sensitivities of the hindpaws or forepaws were assessed using repeated applications of graded von Frey filaments (0.04–2 g) forces to the plantar surface of the paw. Withdrawal frequencies (presented as %) and 40% withdrawal thresholds (filament force which elicited 40% or more withdrawal responses) were calculated from five applications per filament. Experimental groups for optogenetic manipulation were tested without optical stimulation at baseline and with optical stimulation for differences in mechanical sensitivity. The bulk of experiments were performed with the H134R variant of ChR2, which is the version conventionally used, and also characterized extensively by us in the cortex^49^. However, we were concerned whether it would be sufficiently fast enough to enable high frequency gamma activity (i.e., 80 Hz). The E456T variant was therefore tested in some experiments in order to evoke action potentials at higher frequencies (>40 Hz) more reliably compared to the H134R variant. In our experiments, however, we also observed 80 Hz gamma rhythms with the H134R variant. Furthermore, animals expressing either the H134R or E149T variants did not show any differences in mechanical sensitivity at baseline or during stimulation, hence behavioral measurements from the two groups were pooled. Measurements were taken by a researcher blinded to the identity of the animals.

For the study of the descending pathways, von Frey baseline mechanical testing was performed before an acute non-invasive intrathecal injection of 2 mM granisetron hydrochloride (10 µl, dissolved in saline; Tocris) or sterile saline injection, which was given under a brief 1% isoflurane anesthesia as previously described^65^. This procedure involves locating the prominent spinous process of L6 with a gentle press and carefully inserting the needle (30G attached to a Hamilton syringe) between the grooves of the L5 and L6 vertebra. A tail flick during needle insertion indicates successful entry of the needle in the intradural space. Animals that did not display the tail flick were not used for the experiment. Thirty minutes after the injection, a second round of von Frey measurements was taken during laser stimulation (40 Hz, 3 ms pulses, 30 mW mm^−2^). A third behavioral session was performed in these animals one week after the stimulation. Subsequently, the animals received an intrathecal injection of saline (if they had received granisetron previously) or granisetron (if treated with saline previously). The experimenter taking the measurements was always blinded to both the identities of the animals (YFP- or ChR2-expressing) and to the drug (saline or granisetron) that was injected.

In the real-time optogenetic place aversion test, the set-up consisted of two chambers (15 × 15 cm each) that were connected via a neutral middle chamber (8 × 8 cm) and separated by removable doors. Each chamber contained distinct visual and odor cues, with either horizontal or vertical stripes on the chamber walls, along with odor cues (either a cocoa or berry scent). Mice were attached to the optical patch cables and initially placed in the middle neutral chamber. Immediately after the removal of the chamber doors, video recording (by a camera placed above the set-up) of the animal’s movements was started and mice were allowed to freely move in the whole apparatus during baseline sessions. Animals were acclimatized to the apparatus twice a day for five baseline sessions of 20 min each. The chamber preferred (determined by time spent in each chamber) during the last baseline session was paired with the optogenetic light illumination in two subsequent conditioning sessions (20 min each) over 2 consecutive days. During conditioning, animals had free access to all chambers and a 40 Hz illumination (3 ms pulses, 30 mW mm^−2^) was switched on automatically whenever the animal entered the chamber, which it preferred during the last baseline recording. The illumination was immediately turned off once the animal exited the chamber. For analysis, the total time spent in the preferred chamber during the last baseline recording was compared to the total time spent in the same chamber during the last conditioning session. Recording and tracking analysis were performed with the ANY-maze software (Stoelting Co., Ireland). Additionally, the motor function (mean speed over 20 min recording) of the animals during photo-illumination was assessed from the tracking analysis.

In the attention test, a cohort of PV-ChR2-YFP and PV-YFP mice (*n* = 8 per group) implanted unilaterally with optical fibers in the S1HL was trained in the 5-choice serial reaction time (5-CSRT) task using automated Bussey-Saksida Mouse Touch Screen operant chambers (Campden Instruments, Loughborough, UK) and ABET II TOUCH software (Lafayette Instrument, IN, USA). Throughout training and testing stages, animals had limited access to drinking water (30 min per day) and hence water could be used as a reward to reinforce correct choice behavior in individual trials during the task. For habituation and training, procedures outlined in Humby et al.^29^ and the ABET II TOUCH 5-CSRT task module (version 3) were followed. Briefly, a light cue was presented in one out of five windows for a given time period and a trial was counted as correct if the animal touched the monitor of the cued window within an extended time of 5 s after the cue disappeared. If the mouse interacted with another window (incorrect trial) or no touch screen interaction was detected (omission trial) following cue presentation a punishing time out period was signaled by the house light turning on for 5 s. A session consisted of 60 trials and mice performed one session per day. During training the optical patch cable was connected daily with the laser turned off, and the cue duration was successively reduced from 30 to 2 s. Once performance at 2 s cue presentation reached a criterion of >80% accuracy [number of correct trials/total number responded trials (correct + incorrect)] and < 20% omissions [number of missed trials/number of trials presented] for 2 consecutive days, animals were tested on the following day again with a cue duration of 2 s and the laser turned on (40 Hz illumination, 3 ms pulses with an intensity of 30 mW mm^−2^).

### Histology and immunohistochemistry

At the end of the experiment, mice were killed with an overdose of carbon dioxide and transcardially perfused with phosphate-buffered saline (PBS) followed by 4% paraformaldehyde (PFA). Brain tissues were collected and post-fixed additionally for 24 h. Brain sections were cut with a vibratome at 50 µm thickness, mounted with Mowiol and imaged with a fluorescent microscope to confirm the location of AAV injection by YFP expression.

For co-localization immunostaining, anti-parvalbumin (mouse, 1:1000; Millipore; #MAB1572), which has been previously validated for specificity^66^ was used. The anti-Fos (rabbit; Synaptic Systems; #226003) and anti-serotonin (goat; Abcam; #ab66047) were used at 1:5000 and 1:500, respectively. Briefly, sections were incubated in PBS/50 mM glycine for 10 min, followed by a blocking step of 60 min in 7% horse serum with 0.2% Triton in PBS. Sections were incubated in anti-Fos in the blocking solution overnight at 4 °C. The sections were subsequently washed in 7% horse serum in PBS (two 10 min washes) and incubated with secondary antibody (donkey anti-rabbit Alexa 488 or 647; 1:700 each; Thermo Fisher Scientific; #A-21206 and #A-31573, respectively;) in washing solution for 1 h at room temperature. Tissues were washed in PBS twice, incubated in Hoechst (1:10,000 in PBS; Thermo Fisher Scientific; #H3670) for 10 min, washed in PBS and further incubated for 10 min in 10mM TRIS-HCl before mounting. A laser-scanning confocal microscope (Leica TCS SP8, Germany) was used to visualize immunofluorescence levels of the sections. Z-stack images (scanned at 2 µm-thick planes) were taken using identical illumination exposure parameters for sections prepared from YFP and ChR2 animals. Stacked images taken were maximally projected and subsequently overlaid with the corresponding atlas section^67^ to anatomically define the regions of interest for quantification. The ImageJ software (version 1.50a, National Institutes of Health, USA) and Leica Application Suite X (Leica, Germany) were used to visualize and to stereologically count positive-labeled cells within the boundaries of the defined regions. Experimenters were blinded to the identity of sections they were analyzing. Specificity of the antibody staining was tested by omitting the primary antibody, in which no immuno-positive labeling was found (examples are shown in Fig. 9c and Supplementary Fig. 5a).

For the functional mapping of activated brain regions based on Fos immunostaining, both the YFP- and ChR2-expressing groups of mice were optically illuminated at 40 Hz (same conditions as in behavior tests above) for a total of 20 min (with a 3 min pause after 10 min) 1 h before perfusion.

### Statistical analysis

All data are expressed as mean ± standard error of the mean (S.E.M.) unless stated otherwise. Prism (version 7.03) was used for all statistical analysis. Two-Way ANOVA with repeated measures for both factors and Bonferroni’s multiple comparison test between factors or for differences relative to the pre-stimulation baseline within a factor were performed for the binned time-frequency power analyses. A one-way ANOVA with Tukey’s multiple comparison test was used to assess withdrawal latency differences between von Frey filaments and pin-prick stimuli. All withdrawal frequencies were compared using the two-way ANOVA for repeated measures with Bonferroni’s post-hoc test for multiple comparisons. A paired *t*-test was used to compare withdrawal thresholds and cumulative effects in ERP power over the duration from 0 to 2 s following stimulation. In the case of a failed normality test, the Wilcoxon-signed rank test was applied. The Mann–Whitney rank sum test was applied for the Fos-positive counts. In all tests, a *p*-value of < 0.05 was considered significant.

### Reporting summary

Further information on experimental design is available in the Nature Research Reporting Summary linked to this article.

## Supplementary information

Supplementary Information

Reporting Summary

## Data Availability

The raw data that support the findings of this study are available from the corresponding author upon request.
