# Supplementary material for: Gamma oscillations in somatosensory cortex recruit prefrontal and descending serotonergic pathways in aversion and nociception
Source: Nat Commun. 2019 Feb 28;10:983. doi: 10.1038/s41467-019-08873-z (PMC6395755; doi:10.1038/s41467-019-08873-z)
Supplement: Supplementary file 1 — Supplementary Information [file 41467_2019_8873_MOESM1_ESM.docx]

**Supplementary Information**

**Gamma oscillations in somatosensory cortex recruit prefrontal and descending serotonergic pathways in aversion and nociception**

Linette Liqi Tan*, Manfred Josef Oswald*, Céline Heinl*, Oscar Andrés Retana Romero, Sanjeev Kumar Kaushalya, Hannah Monyer, Rohini Kuner

**Supplementary Figures**

**
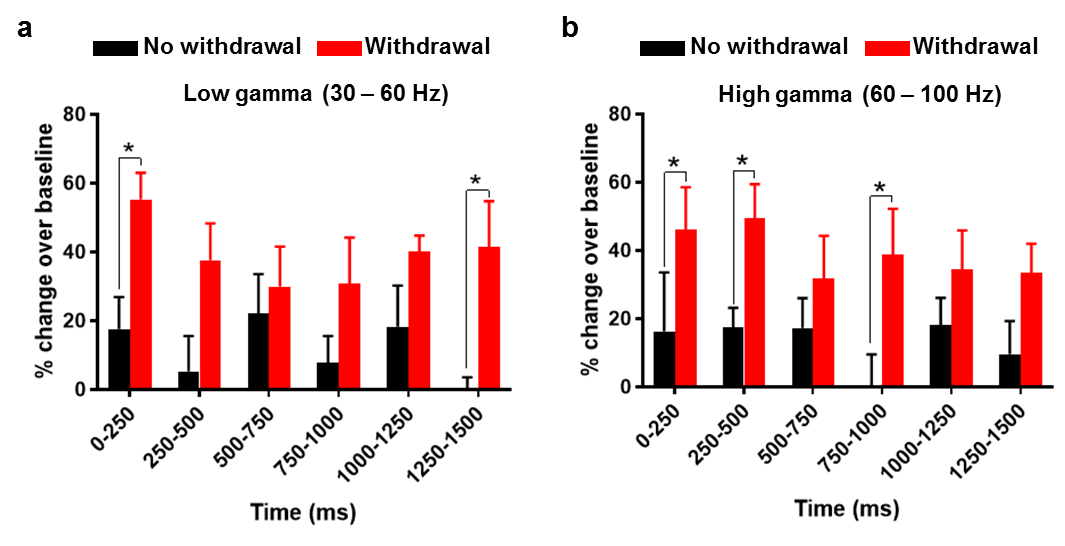
**

**Supplementary Figure 1:** Time course of **(a)** low and **(b)** high gamma frequency power changes (normalised to baseline) in the S1HL of naive mice in the absence or presence of paw withdrawals to mechanical stimulation with a 2 g von Frey filament (*n = 7* in all panels) over the duration of 1.5 s immediately after filament application (left panels). Data are represented in 250 ms time bins (averaged first over all withdrawal and all no withdrawal trials for each animal) and are plotted as mean ± standard error of mean across animals; **p*<0.05 compared between groups, two-way repeated measures ANOVA with Bonferroni multiple comparison test.

**
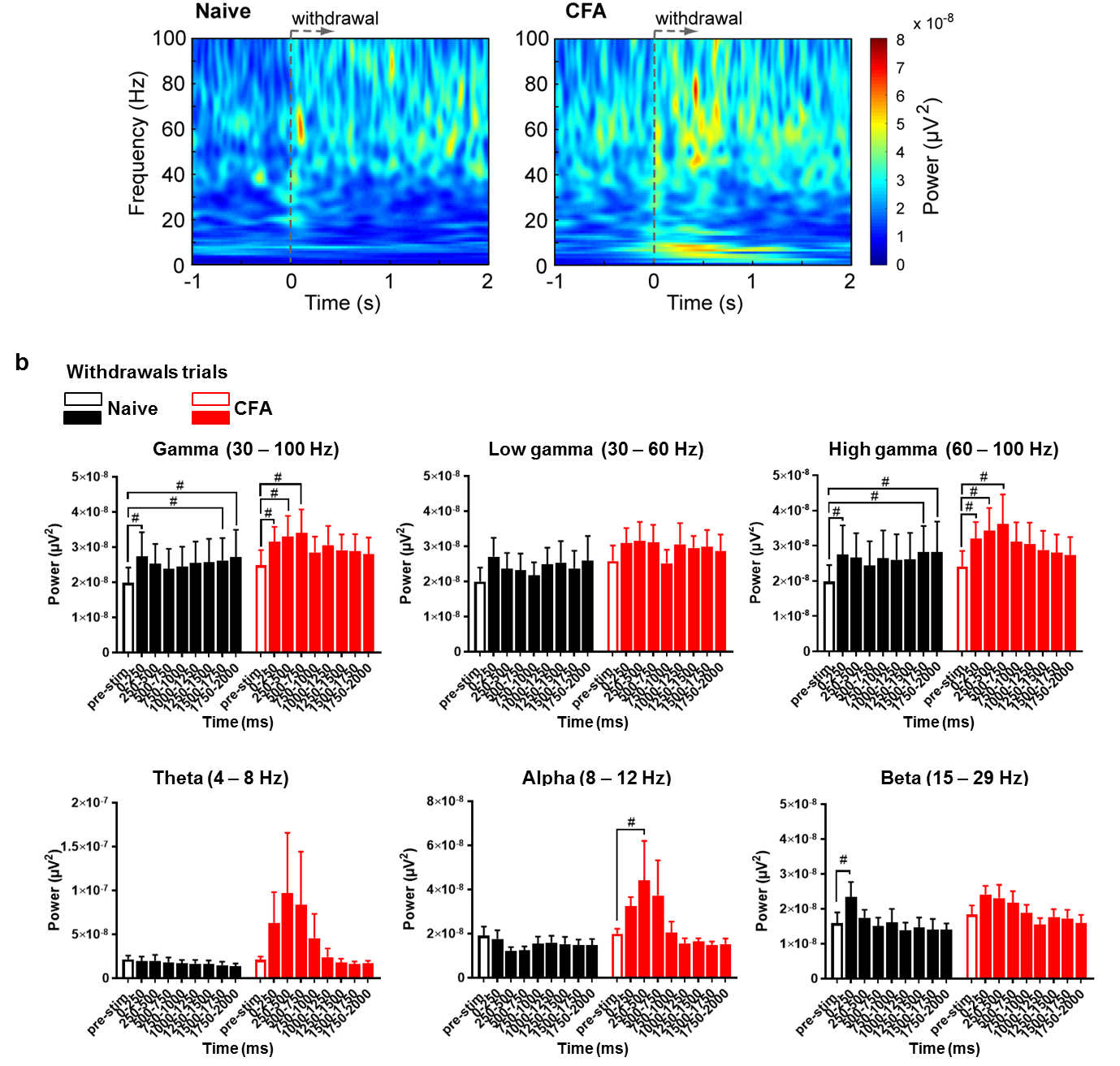
**

**a**

**Supplementary Figure 2: (a)** Time frequency representation of spectral modulation in S1HL of naive (left) and CFA-inflamed (right) mice during paw withdrawal trials in response to 2 g von Frey stimulation of the contralateral hindpaw aligned at stimulus onset (grand mean, *n = 7* animals, 5 – 7 applications per filament and animal). **(b)** Quantification of frequency power in paw withdrawal trials evoked by 2 g von Frey filament application over the 2 s post-stimulation period (represented in 250 ms time bins) in the S1HL of naive and CFA-inflamed mice (*n = 7* in all panels). Data are represented as mean ± standard error of mean; ^#^*p*<0.05 compared to pre-stimulation values of respective group, two-way repeated measures ANOVA with Bonferroni multiple comparison test.

**
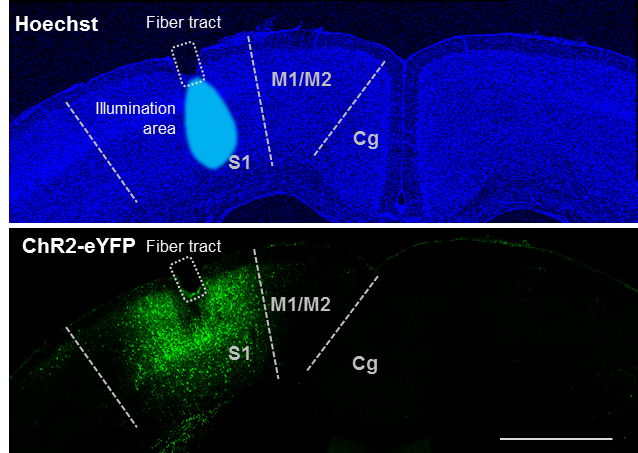
**

**Supplementary Figure 3:** Representative images of Hoechst nuclei staining (blue, upper panel) and ChR2-eYFP (green, lower panel) expression in the S1 cortex. Expression of ChR2-eYFP was not detected in the adjacent motor cortex (M1/M2). The dotted outline in both panels indicate the fiber tract (site of implantation). The blue shaded region in the upper panel indicates the estimated depth of illumination based upon the maximum light intensity (~ 30 mm mm^-2^) used. Scalebar, 1 mm.


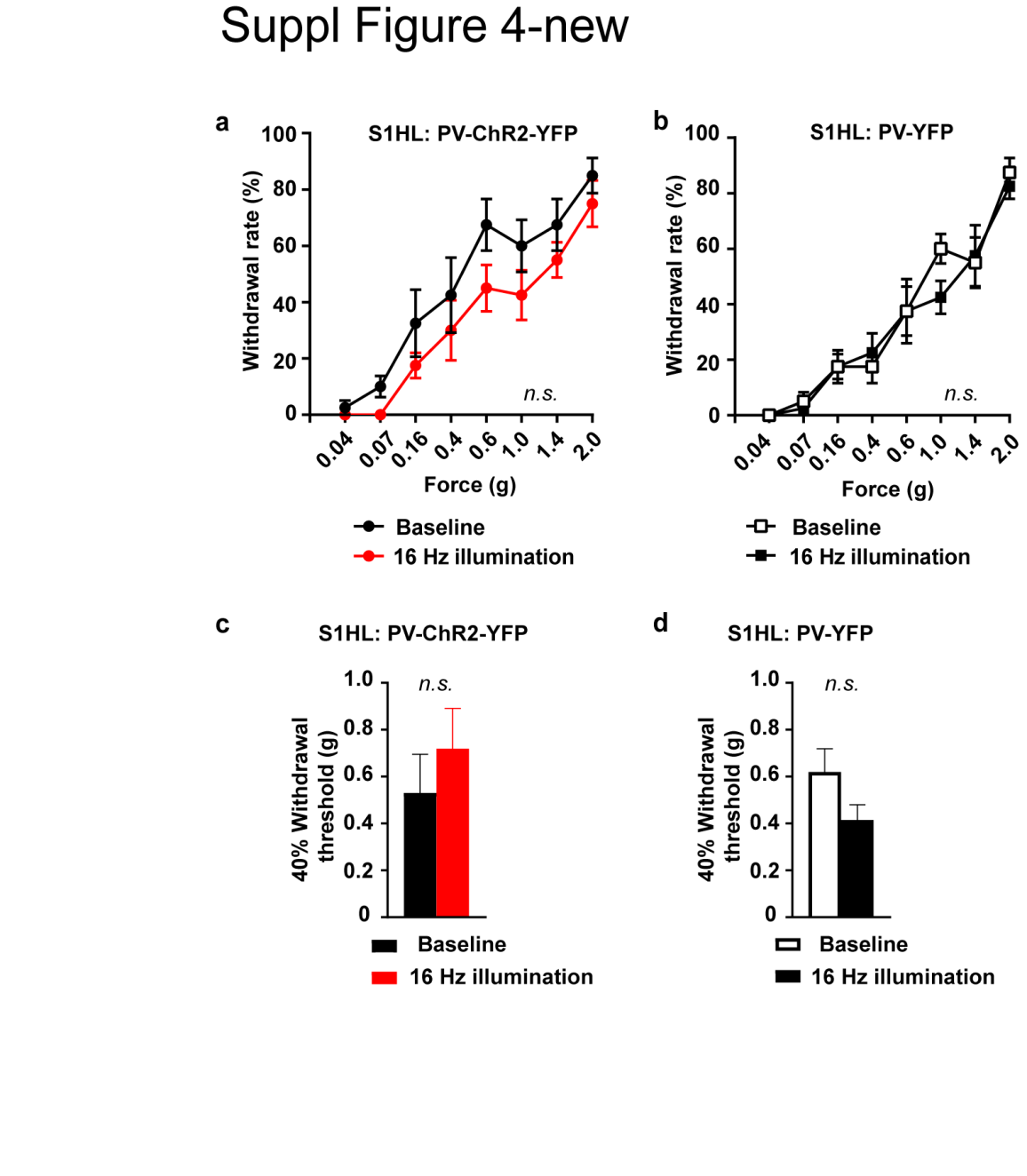


**Supplementary Figure 4:** Effects of 16 Hz illumination of the S1HL cortex of PV-CHR2-YFP and PV-YFP mice on hindpaw withdrawal responses to von Frey filament applications. **(a, b)** Withdrawal frequency rates do not differ between baseline and during 16 Hz illumination in PV mice expressing ChR2 or YFP mice (both panels, *n* = 7). **(c, d)** The 40% mechanical thresholds are not significantly different between baseline and 16 Hz illumination of these PV-ChR2-YFP and PV-YFP mice (both panels, *n* = 7). Data are represented as mean ± standard error of mean. *p*>0.05, two way repeated measures ANOVA in panels a and b, paired Student’s *t*-test in panels c and d; *n.s.*, not significant.


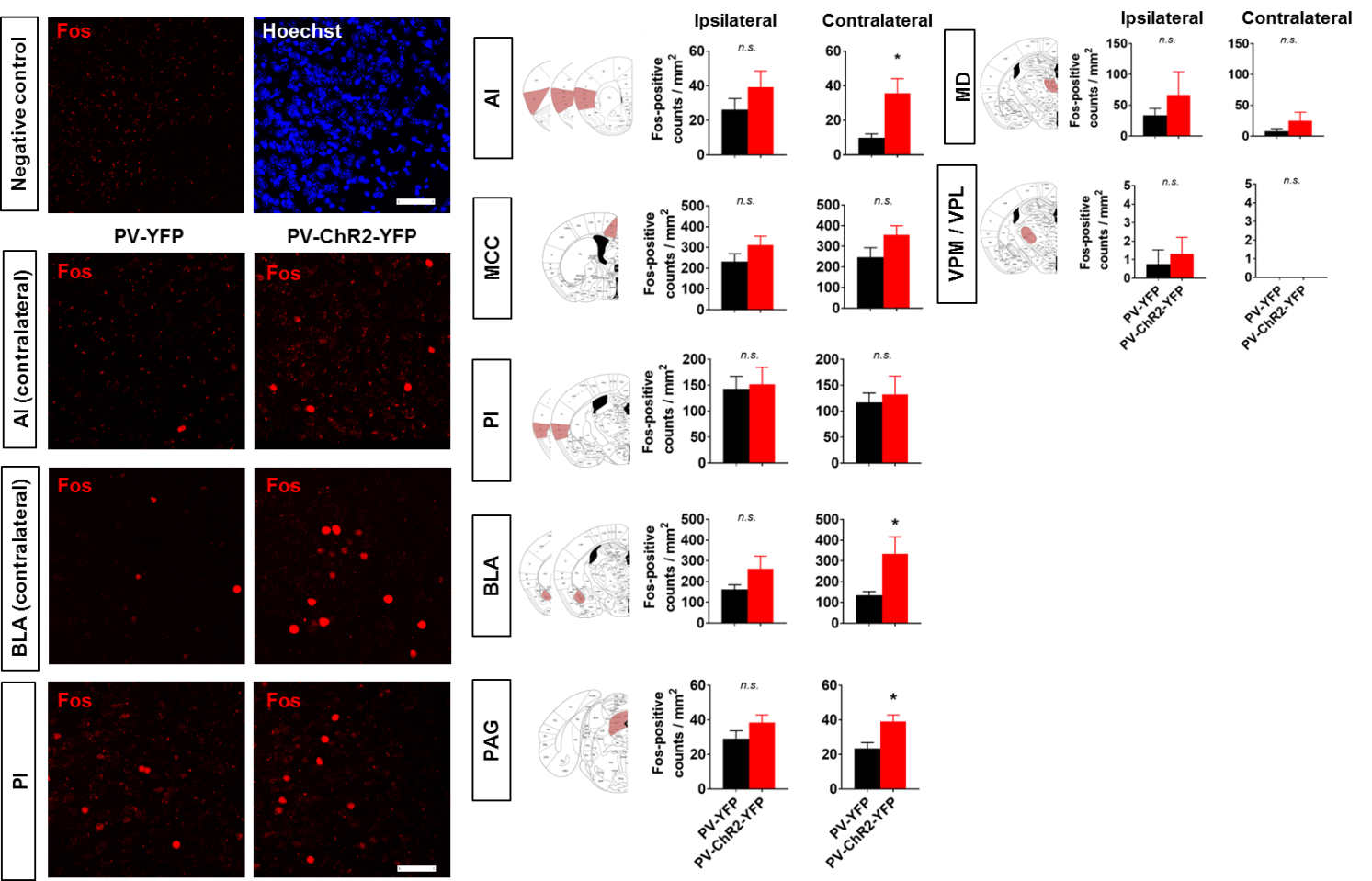


**Supplementary Figure 5:** Functional mapping of Fos upregulation activity following optogenetically-induced 40 Hz gamma activity in the S1HL cortex in PV-YFP and PV-ChR2-YFP mice. **(a)** An example image of negative control for anti-Fos staining (red, top left) and nuclei staining shown with Hoechst (blue, top right). Example images of Fos expression in various brain regions from control PV-YFP and PV-ChR2-YFP mice following 40 Hz entrainment in the S1HL are shown in the lower panels. Scale bars represent 50 µm. **(b)** Quantification of Fos-positive counts are shown for the ipsilateral and contralateral brain regions in PV-YFP (*n* = 6 – 8 per brain region) and PV-ChR2-YFP (*n* = 7 – 9 per brain region). Abbreviations: anterior insula (AI), basolateral amygdala (BLA), posterior insula (PI), mid-cingulate cortex (MCC), periaqueductal gray (PAG), medial thalamus (MD), ventral posterolateral thalamus (VPL), ventral posteromedial thalamus (VPM). Data are represented as mean ± standard error of mean. **p*<0.05, Mann-Whitney rank sum test; *n.s.*, not significant.
