# Supplementary material for: Gamma oscillations in somatosensory cortex recruit prefrontal and descending serotonergic pathways in aversion and nociception
Source: Nat Commun. 2019 Feb 28;10:983. doi: 10.1038/s41467-019-08873-z (PMC6395755; doi:10.1038/s41467-019-08873-z)
Supplement: Supplementary file 2 — Reporting Summary [file 41467_2019_8873_MOESM2_ESM.pdf]

nature research

Corresponding author(s): Rohini Kuner
NCOMMS-17-03925B-Z

Last updated by author(s): Jan 19, 2019

Reporting Summary

Nature Research wishes to improve the reproducibility of the work that we publish. This form provides structure for consistency and transparency in reporting. For further information on Nature Research policies, see Authors & Referees and the Editorial Policy Checklist.

Statistics

For all statistical analyses, confirm that the following items are present in the figure legend, table legend, main text, or Methods section.

n/a	Confirmed	
☐	☒	The exact sample size (*n*) for each experimental group/condition, given as a discrete number and unit of measurement
☐	☒	A statement on whether measurements were taken from distinct samples or whether the same sample was measured repeatedly
☐	☒	The statistical test(s) used AND whether they are one- or two-sided<br>*Only common tests should be described solely by name; describe more complex techniques in the Methods section.*
☐	☒	A description of all covariates tested
☐	☒	A description of any assumptions or corrections, such as tests of normality and adjustment for multiple comparisons
☐	☒	A full description of the statistical parameters including central tendency (e.g. means) or other basic estimates (e.g. regression coefficient) AND variation (e.g. standard deviation) or associated estimates of uncertainty (e.g. confidence intervals)
☐	☒	For null hypothesis testing, the test statistic (e.g. *F*, *t*, *r*) with confidence intervals, effect sizes, degrees of freedom and *P* value noted<br>*Give P values as exact values whenever suitable.*
☒	☐	For Bayesian analysis, information on the choice of priors and Markov chain Monte Carlo settings
☒	☐	For hierarchical and complex designs, identification of the appropriate level for tests and full reporting of outcomes
☐	☒	Estimates of effect sizes (e.g. Cohen's *d*, Pearson's *r*), indicating how they were calculated

Our web collection on statistics for biologists contains articles on many of the points above.

Software and code

Policy information about availability of computer code

Data collection	Leica Application Suite X (Leica, Germany), ANY-maze software (Stoelting Co., Ireland), ABET II TOUCH software (Lafayette Instrument, IN, USA), Neuralynx (Neuralynx, Inc, USA)
Data analysis	Microsoft Excel 2010, Image J (version 1.50a, National Institutes of Health, USA), Leica Application Suite X (Leica, Germany), Prism (version 7.03), Brainstorm(http://neuroimage.usc.edu/brainstorm), Matlab (The Mathworks Inc.MA, USA)

For manuscripts utilizing custom algorithms or software that are central to the research but not yet described in published literature, software must be made available to editors/reviewers. We strongly encourage code deposition in a community repository (e.g. GitHub). See the Nature Research guidelines for submitting code & software for further information.

Data

Policy information about availability of data

All manuscripts must include a data availability statement. This statement should provide the following information, where applicable:

- Accession codes, unique identifiers, or web links for publicly available datasets
- A list of figures that have associated raw data
- A description of any restrictions on data availability

The raw data that support the findings of this study are available from the corresponding author upon request.

Field-specific reporting

Please select the one below that is the best fit for your research. If you are not sure, read the appropriate sections before making your selection.

☒ Life sciences ☐ Behavioural & social sciences ☐ Ecological, evolutionary & environmental sciences

Life sciences study design

All studies must disclose on these points even when the disclosure is negative.

Sample size	In previous experiments we have determined the sample size using G-power analyses and therefore have a very clear set of what sample size is required for the behavioral and histochemical data reported. The number of animals used in the behavioral experiments was consistent with standards in the field and with our own previous publications (Simonetti et al., 2014; Paldy et al., 2017; Tan et al., 2017). In our electrophysiological experiments, the number of animals we used (n = 11) is fully sufficient according to power analysis and exceeds n numbers seen in other such publications (Lepousez and Lledo, 2013; Iaccarino et al., 2016; Saleem et al., 2017). For example, in a post-hoc Power analysis for the % ERP differences in wide gamma power (Fig. 1c) we calculated an effect size of 1.6 from the ratio of the mean difference to the standard deviation. An a-priori power analysis performed with G-power determines a minimum group size of 7 for a paired t-test comparison with an effect size level of 1.5, an alpha error probability of 0.05, and a Power of 0.95 (1-beta error probability).
Data exclusions	No data points were excluded.
Replication	Each experiments were replicated at least 7 times (n=7-13) and animals were always sampled from more than 2 different litters.
Randomization	Mice were randomly allocated to receive stereotactic injections of either control- or opsin-expressing viral constructs.
Blinding	Investigators were blinded to the identity of the animals and immunostained sections they were measuring for the behavioral tests and Fos expression analysis.

Reporting for specific materials, systems and methods

We require information from authors about some types of materials, experimental systems and methods used in many studies. Here, indicate whether each material, system or method listed is relevant to your study. If you are not sure if a list item applies to your research, read the appropriate section before selecting a response.

Materials & experimental systems

n/a	Involved in the study	
☐	☒	Antibodies
☒	☐	Eukaryotic cell lines
☒	☐	Palaeontology
☐	☒	Animals and other organisms
☒	☐	Human research participants
☒	☐	Clinical data

Methods

n/a	Involved in the study	
☒	☐	ChIP-seq
☒	☐	Flow cytometry
☒	☐	MRI-based neuroimaging

Antibodies

Antibodies used	Antibodies used in this study include rabbit anti-Fos (Synaptic Systems; #226003, 1:5000), mouse anti-parvalbumin (Millipore;#MAB1572, 1:1000), goat anti-serotonin (Abcam; #ab66047, 1:500), donkey anti-rabbit Alexa Fluor 488 (Thermo Fisher Scientific; #A-21206, 1:700 dilution) or donkey anti-rabbit Alexa Fluor 594 (Thermo Fisher Scientific; #A-31573, 1:700 dilution).
Validation	Validation of antibodies are provided in Fig. 9c ad Supplementary Fig 4a, and cited in the Methods.

Animals and other organisms

Policy information about studies involving animals; ARRIVE guidelines recommended for reporting animal research

Laboratory animals	Adult (four to eight month old) male and female Parvalbumin-Cre mice with a C57BL/6 background were used.
Wild animals	This study did not involve wild animals.
Field-collected samples	This study did not involve field-collected samples.
Ethics oversight	All experimental procedures were performed according to the ethical guidelines set by the local governing body (Regierungspräsidium Karlsruhe, Germany; approval numbers 35-9185.81/G119/14 and 35-9185.81/G184/18).

Note that full information on the approval of the study protocol must also be provided in the manuscript.
